# Soil dissolved organic matter dynamics under dryland restoration: disentangling land use, vegetation, and management controls

**DOI:** 10.3389/fpls.2026.1934903

**Published:** 2026-08-26

**Authors:** Yi-cong Wang, Qing-wei Zhang, Nan-yan Li, Yu-hui Zhang, Guo Chen, Dong-xu Wu, Guo-xiong Gao, Qiang Zhang, Feng-bao Zhang, Jian Wang, Ming Li, Hao Wang

**Affiliations:** 1 State Key Laboratory of Soil and Water Conservation and Desertification Control, College of Soil and Water Conservation Science and Engineering, Northwest A&F University, Yangling, Shaanxi, China; 2 College of Urban and Environmental Sciences, Peking University, Beijing, China; 3 College of Natural Resources and Environment, Northwest A&F University, Yangling, Shaanxi, China

**Keywords:** dissolved organic matter (DOM), land use, management intensity, optical indicators, vegetation traits

## Abstract

**Introduction:**

Dryland soils are highly sensitive to land use change and restoration, yet the relative influence of land use and associated vegetation and management conditions on soil dissolved organic matter (DOM) remains poorly understood.

**Methods:**

This study evaluated DOM quantity and quality across five land use types (cropland, orchard, grassland, shrubland and forestland) and the additional differences associated with vegetation and management within specific land use systems. Soils were sampled from sixteen sites and three depths (0-20, 30-50, and 60-80 cm), including naturally restored and artificially planted shrublands, grasslands with taproot and fibrous-root systems, orchards under high-input and low-input management.

**Results:**

Dissolved organic carbon (DOC) and DOM optical properties were characterized using ultraviolet-visible absorbance and fluorescence spectroscopy. Overall, DOC content was highest in forestland topsoil (235.22 mg kg^−1^) and lowest in orchards and cropland (10.16-89.15 mg kg^−1^), and declined with depth. Humic-like components dominated the fluorescence signal, whereas protein-like components increased in deeper layers. Woody systems showed higher optical signatures consistent with larger apparent molecular size, greater aromaticity, and stronger humification, whereas cropland showed the opposite pattern. Within land use types, the walnut/low-input orchard showed higher DOC and stronger humic-like optical signals than the apple/high-input orchard, whereas grassland root type and shrubland restoration mode showed weaker changes. Multivariate analysis showed that DOC content and DOM composition covaried with surface inputs, fine texture, and biological soil crust development.

**Discussion:**

This framework provides a useful basis for interpreting soil carbon responses to restoration and land management in fragile dryland ecosystems.

## 1 Introduction

Drylands occupy approximately 41% of the global land surface, and their ecosystem functioning is highly vulnerable to climate change and human disturbance (Prăvălie, 2016). Under such sensitivity, relatively small changes in soil carbon pools can induce shifts in ecosystem services and biogeochemical regulation at regional to even global scales (Bolan et al., 2011; Schimel, 2010). Land use change and management practices often interact in dryland landscapes, making it necessary to consider their combined influence when evaluating soil carbon cycling (Helms et al., 2008; Prăvălie, 2016). Soil organic carbon (SOC) is widely adopted as a primary indicator of soil quality, fertility maintenance, and carbon sequestration potential (Bolan et al., 2011). Yet SOC responses to land use and management are often inconsistent in both direction and magnitude (Beillouin et al., 2023; Kaiser and Kalbitz, 2012). One reason is that bulk SOC integrates multiple organic matter fractions with contrasting turnover rates, accessibility, and persistence mechanisms. Therefore, bulk SOC alone is often insufficient for diagnosing short-term carbon dynamics or assessing management regulation (Lavallee et al., 2020; Leinemann et al., 2018; Naipal et al., 2018).

Among soil organic matter fractions, dissolved organic matter (DOM) is one of the most mobile and biogeochemically reactive pools, and plays an important role in carbon cycling (Kothawala et al., 2021; Ren et al., 2024). It originates mainly from litter leaching, root exudation, and microbial metabolites, and comprises a chemically diverse mixture of compounds, including humic-like and protein-like substances rich in reactive functional groups (e.g., carboxyl and phenolic hydroxyl moieties), which confer high reactivity during sorption, complexation, and biological transformation (Bolan et al., 2011; Speetjens et al., 2022). Owing to its mobility and compositional heterogeneity, DOM is closely involved in microbial metabolism, as well as transport and complexation of nutrients and contaminants. DOM also responds rapidly to shifts in vegetation input, soil properties, and management, making it particularly informative for tracing active carbon dynamics and profile-scale biogeochemical processes (D'Andrilli et al., 2022; Schimel, 2010). Accordingly, DOM quantity and quality have been increasingly regarded not only as sensitive indicators of soil functional status and biogeochemical responses to environmental variation, but also as promising proxies for ecosystem functioning and, in some cases, persistence-related behavior of soil organic matter itself (Aiken et al., 2011; Tian et al., 2025).

Methodologically, DOM is widely characterized using ultraviolet–visible (UV–Vis) absorbance and fluorescence spectroscopy. Optical indices such as specific UV absorbance at 254 nm (SUVA_254_) and the absorbance slope ratio (S_R_) are commonly used to infer aromaticity and molecular-weight–related properties (Ohno, 2002; Weishaar et al., 2003). Fluorescence excitation–emission matrices (EEMs) combined with parallel factor analysis (PARAFAC) can identify humic-like and protein-like fluorescent components within DOM. Complementary indices (i.e., the fluorescence index (FI), humification index (HIX), and biological index (BIX)) further support interpretations of DOM sources and humification status (Gao et al., 2017; Rinot et al., 2021). Therefore, these optical metrics enable integrated assessment of DOM composition, quality, and their underlying controls, thereby complementing bulk SOC measurements (Han et al., 2024; Teixeira et al., 2024).

Land use change can reconfigure DOM dynamics by reshaping vegetation composition, litter inputs, and the soil microenvironment. These effects may vary among restoration pathways and land-use transitions and may persist across the soil profile (Han et al., 2023; Weishaar et al., 2003). In dryland regions of the Loess Plateau, long-term cultivation has been widely replaced by or converted into grassland restoration, shrub establishment, forest development, and orchard planting (Chen et al., 2023; Xu et al., 2018). These pathways may differ not only in the amount of organic carbon returned to soil, but also in the chemical nature and vertical distribution of organic inputs and their subsequent biological transformation into DOM (Liu et al., 2015). Nevertheless, most existing studies have emphasized broad differences among land use types, whereas the additional effects of vegetation characteristics and management practices within specific land use systems remain comparatively underexplored (Chen et al., 2024; Zethof et al., 2019). For example, high-input versus low-input orchard management can alter litter return, soil disturbance, nutrient inputs, and surface crust development, thereby jointly regulating DOM quantity and quality (Beillouin et al., 2023; Peng et al., 2023). In grassland systems, contrasting root architectures, such as taproot and fibrous-root systems, may influence root-derived carbon inputs and rhizosphere processing, leading to divergence in DOM composition and retention-related behavior (Peixoto et al., 2020; Peng et al., 2023; Wang et al., 2024). In shrublands, natural succession versus artificial planting can modify understory structure, resource competition, and shrub adaptation, with cascading consequences for organic inputs, rhizosphere processes, and soil properties, thereby altering DOM characteristics (Zhang et al., 2025). Therefore, beyond comparisons among land use types, incorporating variables within land use types, such as orchard management intensity, grassland root type, and shrubland restoration mode, can provide a more mechanistic understanding of DOM divergence during dryland restoration and land-use transitions (Chen et al., 2024; Weishaar et al., 2003; Wilson and Xenopoulos, 2009).

Depth-dependent behavior is a defining feature of soil DOM. As DOM moves through the soil profile, its properties often change markedly with depth, and contrasts among soils may become more pronounced in deeper horizons. This means that sampling restricted to topsoil may underestimate subsoil physicochemical controls on DOM dynamics and function (Charamba et al., 2025; Tian et al., 2025). Biological soil crusts (BSCs), plant roots and litter influence DOM inputs and export by regulating aggregate stability, nutrient cycling, and near-surface hydrology, which in turn shape the redistribution of DOM from the soil surface into deeper layers of the soil profile (Riveras-Muñoz et al., 2022; Szyja et al., 2023). From a carbon-budget perspective, DOC leaching represents a recognized but often underestimated terrestrial carbon flux, whose magnitude and vertical expression are jointly regulated by hydrological transport and external inputs, resulting in divergent DOM responses between topsoil and subsoil and underscoring soil depth as a critical dimension for interpreting DOM change (Hu et al., 2024; Nakhavali et al., 2020). Moreover, mineral-surface interactions and microbial transformation regulate DOM retention, release, and processing across mineral-associated and aggregate-protected domains, thereby shaping DOM quantity and quality along the profile (Leinemann et al., 2018). However, most studies have focused either on topsoil or on vertical patterns within a single land use type, whereas integrated assessments that simultaneously consider land use type, restoration pathway, vegetation traits, soil depth, and management intensity within a unified field framework remain limited (Charamba et al., 2025; Li et al., 2019; Ren and Cai, 2025). Few field studies have combined a profile-scale comparison among land use types with embedded within-land-use contrasts under a common climatic and soil-forming setting. This limitation is particularly evident in drylands, where strong moisture limitation, deep rooting, and the juxtaposition of anthropogenic management and natural restoration can amplify profile-scale effects (He et al., 2024; Young et al., 2021).

To address these gaps, we conducted a field comparison in a typical dryland watershed using 16 representative sites that captured broad land use settings together with embedded contrasts in vegetation and management. These contrasts included (1) shrubland under natural restoration (NR) versus artificially planted sites (AP); (2) grasslands dominated by taproot (TR) versus fibrous-root (FR) vegetation; and (3) orchards under high-input management (HM) versus low-input management (LM). Soils were sampled at three depth intervals (0–20, 30–50, and 60–80 cm), and optical techniques were used to characterize soil DOM across soil depth, land use, vegetation, and management, and to explore the underlying mechanisms. We hypothesized that: (i) Compared with cropland, restored woody and grassland systems would have higher DOC content and stronger humic-like and aromatic DOM signatures, reflecting greater organic matter inputs and reduced disturbance; (ii) DOM quantity and composition would show clear vertical differentiation along the soil profile, with declining DOC content and shifts in fluorescence composition with increasing depth; and (iii) vegetation traits, restoration mode, and management intensity would further reshape DOM characteristics within land use types, with distinct manifestations between topsoil and subsoil layers. Collectively, our findings would improve understanding of how dryland restoration pathway, soil depth, and within-system vegetation and management contrasts shape soil DOM dynamics, with implications for evaluating changes in active soil carbon pools under land-use transition.

## 2 Materials and methods

### 2.1 Study area overview and site selection

**Figure 1:**
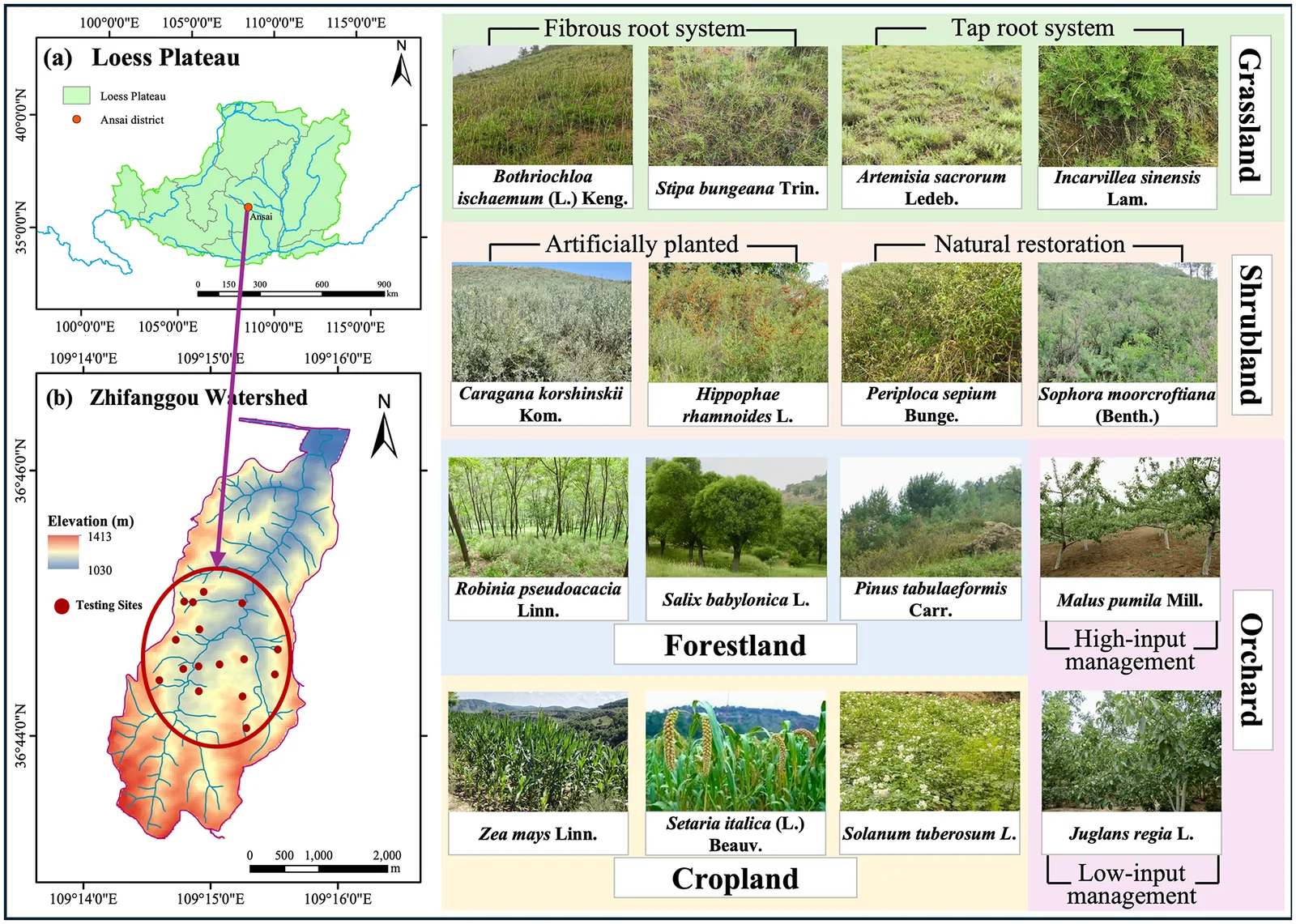
Study area and sampling sites. **(a)** Location of Ansai District within the Loess Plateau; **(b)** elevation and distribution of the 16 sampling sites in the Zhifanggou watershed. Photographs are grouped by grassland root system, shrubland restoration mode, forest and crop species, and orchard species-management categories.

The study was conducted in the Zhifanggou watershed (8.27 km²) located in the typical hilly–gully region of the Chinese Loess Plateau. The watershed is highly dissected, with a gully density of 8.1 km km^-^² (Chen et al., 2023; Zhao et al., 2016). The climate is typically dryland, with a mean annual temperature of 8.8 °C and mean annual precipitation of 505 mm. Precipitation is strongly seasonal and approximately 70% concentrated in June to September (Xu et al., 2018). Soils are dominated by young loess-derived soils developed on aeolian loess, typically silt loam in texture and classified as Typic Ustorthents according to USDA Soil Taxonomy (Messing et al., 2003). Organic matter in the cultivated horizon is generally low (0.53–0.77%) (Xu et al., 2018). Historically, inappropriate land use caused vegetation degradation, whereas soil and water conservation measures since the late 20th century have reshaped land use patterns. Currently, forestland, shrubland, and grassland dominate the watershed and together account for 83% of the area (Chen et al., 2023). The main vegetation includes *Robinia pseudoacacia*, *Populus simonii*, *Hippophae rhamnoides*, *Caragana korshinskii*, and *Artemisia sacrorum* (Chen et al., 2023; Zhang et al., 2026) (**Figure 1**).

Following a watershed-wide reconnaissance of topography and vegetation recovery, five land use types with comparable site conditions were selected from a pool of candidate sites. Each land use type comprised 2–4 vegetation sites differing in root architecture, management intensity, restoration pathway, and functional type, resulting in a total of 16 sites (**Table 1**). Orchard, grassland, shrubland, and forestland sites have experienced approximately 20 years of natural or assisted restoration, whereas sloping cropland remains under conventional local cultivation. Orchard management intensity was classified as a relative field category rather than as a strictly quantified input level. O_MP_ site represented high-input management, characterized by more frequent field intervention and lower surface cover, whereas the O_JR_ represented low-input management, with weaker visible disturbance and greater surface cover. Grassland sites comprised G_BI_ and G_SB_ as representative fibrous-root species, as well as G_AS_ and G_IS_ as representative taproot species. Shrubland sites included S_CK_ and S_HR_ representing long-term managed stands, together with S_PS_ and S_SM_ as typical naturally restored stands. Forestland sites were represented by F_RP_, F_SA_, and F_PT_. Cropland sites included C_ZM_, C_SI_, and C_ST_ as representative crops (**Table 1**).

**Table 1:** Land use type, site code, dominant species, and within-land-use comparison category for the 16 sampling sites.

Land use type	Site code	Dominant species	Within-land-use category
Cropland	C_ZM_	*Zea mays* L.	Crop species
	C_SI_	*Setaria italica* (L.) Beauv.	Crop species
	C_ST_	*Solanum tuberosum* L.	Crop species
Orchard	O_MP_	*Malus pumila* Mill.	High-input management
	O_JR_	*Juglans regia* L.	Low-input management
Grassland	G_BI_	*Bothriochloa ischaemum* (L.) Keng	Fibrous-root
	G_SB_	*Stipa bungeana* Trin.	Fibrous-root
	G_AS_	*Artemisia sacrorum* Ledeb.	Taproot
	G_IS_	*Incarvillea sinensis* Lam.	Taproot
Shrubland	S_CK_	*Caragana korshinskii* Kom.	Artificially planted
	S_HR_	*Hippophae rhamnoides* L.	Artificially planted
	S_PS_	*Periploca sepium* Bunge	Naturally restored
	S_SM_	*Sophora moorcroftiana* (Benth.) Baker	Naturally restored
Forestland	F_RP_	*Robinia pseudoacacia* L.	Tree species
	F_SA_	*Salix babylonica* L.	Tree species
	F_PT_	*Pinus tabulaeformis* Carr.	Tree species

### 2.2 Soil sampling and near-surface measurements

Across the five land use types, a total of sixteen vegetation sites were investigated. At each site, three 50 cm × 50 cm quadrats were established. At each quadrat, a soil pit was excavated, and approximately 0.5 kg of soil was collected from three depth intervals. The topsoil layer (0–20 cm) was strongly influenced by BSCs, litter inputs, and the main distribution of herbaceous roots. The middle soil layer (30–50 cm) was characterized by the presence of shrub roots, with residual effects of litter incorporation. The subsoil layer (60–80 cm) exhibited minimal litter influence and was penetrated only by a limited number of deep woody roots (Chen et al., 2023; Wu et al., 2025). Samples from the three quadrats within each site were composited by depth, sealed in plastic bags, transported to the field station, and air-dried for subsequent analyses.

Near-surface attributes included biological soil crusts thickness (T_BSC_) and coverage (C_BSC_), plant root density (RD), litter density (LD), soil bulk density (BD), soil texture, and vegetation (including understory) cover. T_BSC_ was defined as the thickness of the crust together with its adherent soil layer (Wang et al., 2017a, b; Yair et al., 2011) and was measured ten times using a caliper with 0.03-mm resolution following Wang et al. (2017a). C_BSC_ was first estimated using the quadrat method (Liu et al., 2015) and subsequently refined by digital image analysis of color photographs, during which crust types were recorded (Knapen et al., 2007; Liu et al., 2015). Soil cores for RD and LD were collected using stainless-steel cylinders (10 cm diameter, 5 cm height). Roots and litter were separated by washing through a 1-mm sieve, oven-dried at 65 °C for 12 h, and averaged across sampling points (Wang et al., 2021). Vegetation cover was estimated by three observers in 1 m × 1 m quadrats, and the mean of their estimates was used (Wang et al., 2023a).

### 2.3 DOM extraction and DOC determination

Air-dried soils were gently crushed after removing visible plant residues and gravel, and then passed through a 1-mm sieve to homogenize the material. A total of 48 soil samples were obtained (16 sites × 3 depths), and each sample was processed in quadruplicate. Water-extractable DOM was prepared using ultrapure water. Briefly, 10 g of sieved soil was combined with 100 mL ultrapure water (10:1, v/w) in glass Erlenmeyer flasks, sealed with Parafilm, and shaken at 180 rpm for 60 min at 40 °C. The resulting slurry was centrifuged at 6000 rpm for 6 min, after which the supernatant was filtered through a 0.45-µm membrane to obtain the DOM extract (Jones and Willett, 2006; Li et al., 2019).

To limit photodegradation, the 192 extracts were split into two aliquots and stored in amber vials at 4 °C in the dark. DOC and optical analyses (UV–Vis absorbance and EEM fluorescence) were completed as soon as possible. DOC was measured using a TOC analyzer (TOC-L; Shimadzu, Japan) (Li et al., 2019). Because carbon typically accounts for approximately 67% of organic matter on an elemental basis, DOC was used as a quantitative proxy for DOM concentration in this study (Leinemann et al., 2018; Li et al., 2019).

### 2.4 Spectroscopic analyses

#### 2.4.1 Fluorescence EEMs and PARAFAC modeling

EEMs were acquired on a fluorescence spectrophotometer (RF-6000; Shimadzu, Japan). Excitation wavelengths were scanned from 200 to 500 nm and emission was recorded from 250 to 550 nm, with step sizes of 5 nm (Ex) and 2 nm (Em); the scan speed was 2000 nm min^-^¹. Prior to measurement, extracts were diluted with deionized water to bring DOC below 8 mg L^-^¹, thereby reducing inner-filter artifacts (Cory and McKnight, 2005; Korak et al., 2014). Raw EEMs were processed by subtracting an ultrapure-water blank to correct Raman scatter, masking Rayleigh scattering regions (set to zero), and applying inner-filter correction when necessary, using the corresponding absorbance spectra (Cory and McKnight, 2005; Ohno and Bro, 2006).

PARAFAC modeling was performed in MATLAB using the DOMFluor toolbox (Stedmon and Bro, 2008). Candidate models containing two to seven components were evaluated using loading and residual diagnostics. Split-half analysis was performed with two independent split combinations, and random-initialization analysis used 10 starts. Component congruence between the full-data and split-half solutions was examined using Tucker congruence coefficients. The three-component solution was retained because it was validated in both split combinations and produced stable, interpretable excitation and emission loadings.

Three fluorescence indices were calculated from the corrected EEMs. FI was defined as the emission intensity ratio at 470 nm to 520 nm at Ex = 370 nm, reflecting the relative contributions of autochthonous and allochthonous sources of DOM (Cory and McKnight, 2005). HIX was computed as the ratio of the integrated emission area over 435–480 nm to that over 300–345 nm at Ex = 254 nm and served as an indicator of humification (Ohno, 2002; Wilson and Xenopoulos, 2009). BIX was calculated as the emission ratio at 380 nm to 430 nm at Ex = 310 nm, indicating autochthonous and freshly produced DOM signatures (Huguet et al., 2009).

#### 2.4.2 UV–visible absorbance spectroscopy

UV–Vis absorbance spectra were recorded with a UV-Vis spectrometer (UV-1780; Shimadzu, Japan) using a 10 mm quartz cuvette and ultrapure water as the blank. Absorbance was scanned from 200 to 400 nm at 1 nm intervals (Shafiquzzaman et al., 2014). Specific UV absorbance at 254 nm (SUVA_254_; L mg^-^¹ m^-^¹) was calculated by normalizing absorbance at 254 nm to DOC to indicate aromaticity per unit carbon (Lv et al., 2023; Weishaar et al., 2003). The spectral slope ratio (S_R_), derived from slopes over 275–295 nm and 350–400 nm, was used as a proxy for relative molecular weight (Helms et al., 2008; Li et al., 2014).

### 2.5 Statistical analyses

All variables were tested for normality and homogeneity of variances prior to inferential analyses. When assumptions were met, one-way ANOVA was used to assess differences in DOC content and optical metrics among sites or among depth intervals. Significant effects (*P* < 0.05) were followed by *post hoc* multiple comparisons using Duncan tests. For paired contrasts among vegetation–management factors within the same depth, paired-sample *t*-tests were conducted. Unless otherwise stated, numerical values are reported as mean ± standard deviation (SD), and all figure error bars denote SD.

Pearson correlation analysis and principal component analysis (PCA) were used to summarize co-variation among DOC content, UV–Vis metrics, fluorescence indices, and PARAFAC components. These analyses were used to explore associations between DOM properties and soil physicochemical or near-surface attributes (e.g., texture, BD, RD, LD, vegetation cover, and BSC traits), rather than to infer causal drivers or independent explanatory strength. Before PCA, all included variables were centered and scaled to unit variance. PCA used site-depth means for all 16 sites at the three depths (n = 48), whereas Pearson correlations with surface attributes were restricted to the 16 topsoil observations. All statistical analyses were performed in SPSS 26.0 (SPSS Inc., Chicago, IL, USA), and figures were prepared using Origin 2025b (OriginLab, Massachusetts, USA).

## 3 Results

### 3.1 Variation in soil DOC content

**Figure 2:**
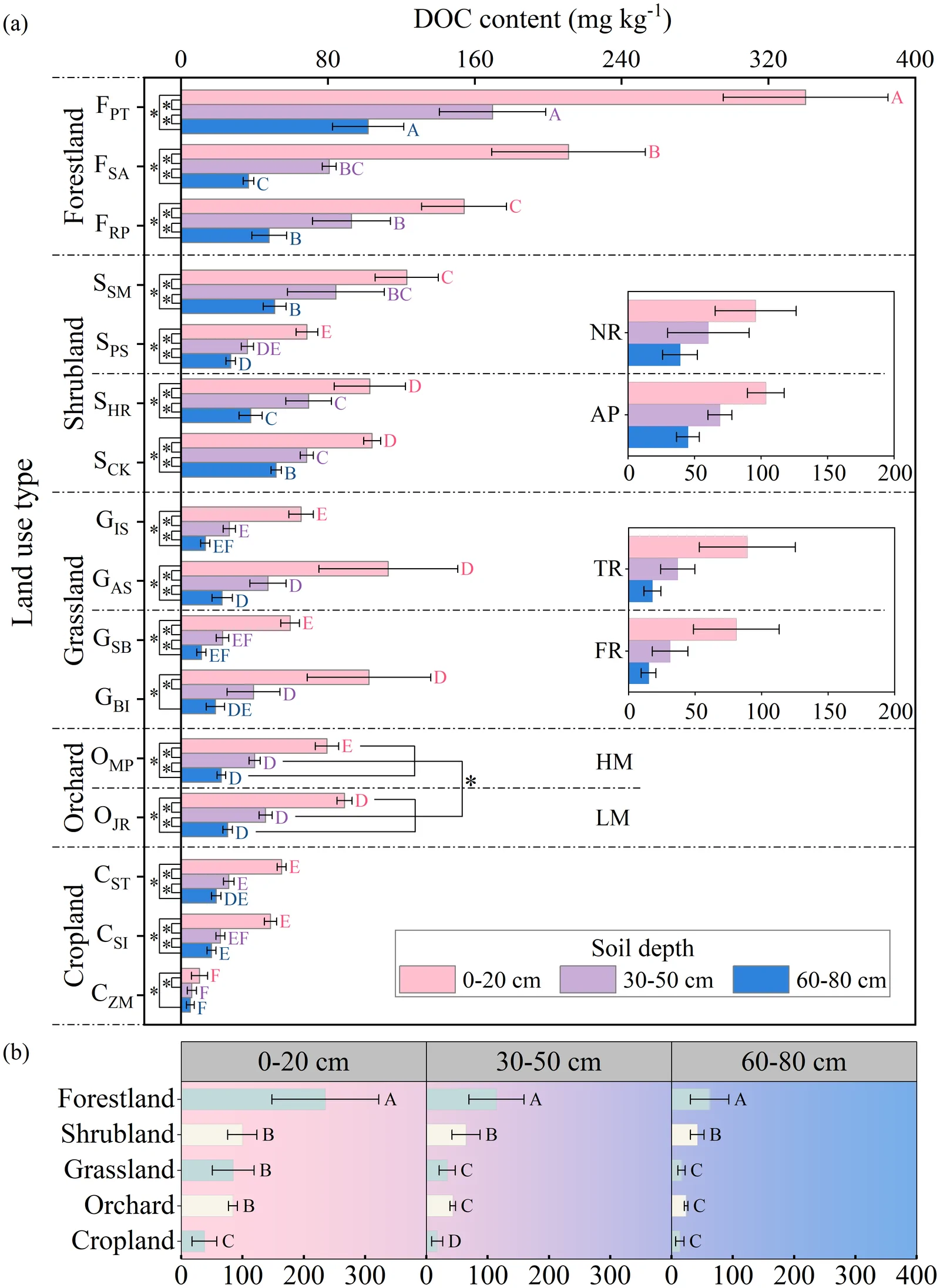
Soil dissolved organic carbon (DOC) content at **(a)** the 16 sampling sites, including within-land-use category summaries, and **(b)** the five land use types at each depth. Pink, purple, and blue bars represent 0–20, 30–50, and 60–80 cm, respectively; error bars denote SD. NR, natural restoration; AP, artificially planted; TR, taproot; FR, fibrous-root; HM, high-input management; LM, low-input management. In panel **(a)**, asterisks beside depth brackets on the left indicate significant differences among depths, and asterisks beside paired-category brackets on the right indicate significant within-land-use contrasts (*P* < 0.05). Within a depth, bars sharing an uppercase letter are not significantly different among sites in panel **(a)** or among land use types in panel **(b)** (*P* ≥ 0.05). Site codes are defined in **Table 1**.

Forestland generally exhibited the highest DOC content, followed by shrubland and grassland, whereas orchard and cropland consistently showed the lowest values. Along the soil profile, DOC content generally declined with depth across all land use types, with significantly higher values in the topsoil than in the middle and subsoil layers. Additional variation was observed among vegetation and management contrasts within several land use types (**Figure 2**).

This overall land-use pattern persisted across the soil profile. In forestland, DOC content at each depth, declining from 235.22 ± 87.05 mg kg^−1^ in the topsoil to 114.47 ± 44.80 and 62.32 ± 31.36 mg kg^−1^ in the middle and subsoil layers, respectively. Orchard and cropland remained lowest (10.16–89.15 mg kg^−1^ in topsoil and generally <30 mg kg^−1^ at depth). Forestland exceeded the other land use types at all depths (P < 0.05). Shrubland, grassland, and orchard did not differ in topsoil, but shrubland exceeded grassland and orchard in the deeper layers (**Figure 2b**).

Further differences emerged within individual land use types. The walnut/low-input orchard site had consistently higher DOC content than the apple/high-input orchard site across all depths (P < 0.05). In cropland, maize had lower DOC content than millet and potato, including a topsoil value of 10.16 ± 4.34 mg kg^−1^. Differences were smaller within shrubland and grassland (**Figure 2a**).

### 3.2 Fluorescence components of soil DOM

**Figure 3:**
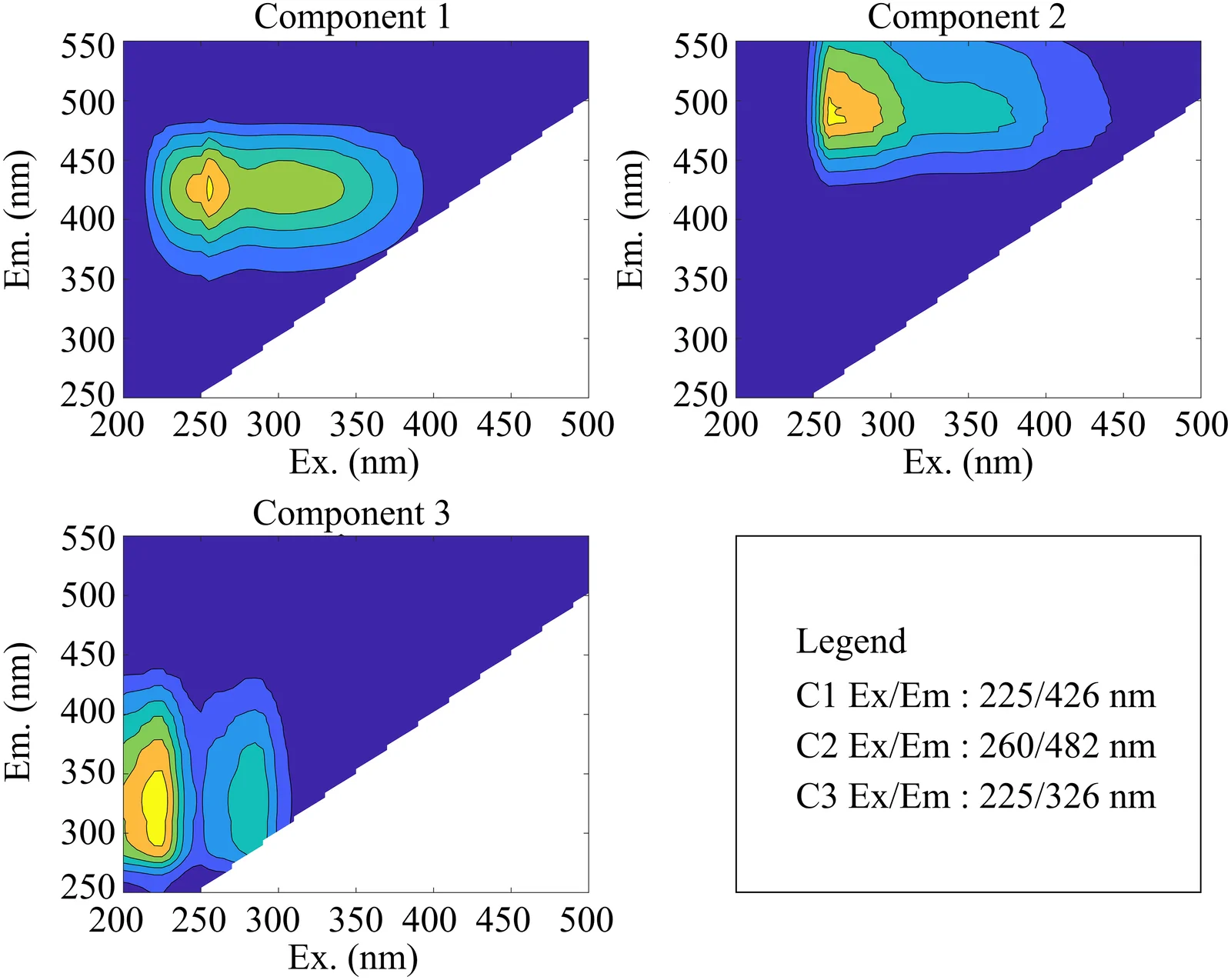
Fluorescence components identified by EEM–PARAFAC analysis: C1, UVA humic-like (Ex/Em = 225/426 nm); C2, terrestrial humic-like (260/482 nm); and C3, protein-like (225/326 nm).

EEM–PARAFAC modeling resolved three fluorescence components across land use types and soil depths. Component C1 was assigned to a UVA humic-like fluorophore, typically associated with higher-molecular-weight and hydrophobic-acid–like fractions (Wang et al., 2023b). Component C2 was commonly regarded as a terrestrial humic-like component (Meilleur et al., 2023). Component C3 represented protein-like material, characterized by tyrosine- and tryptophan-like optical signatures (Williams et al., 2013) (**Figure 3**).

**Figure 4:**
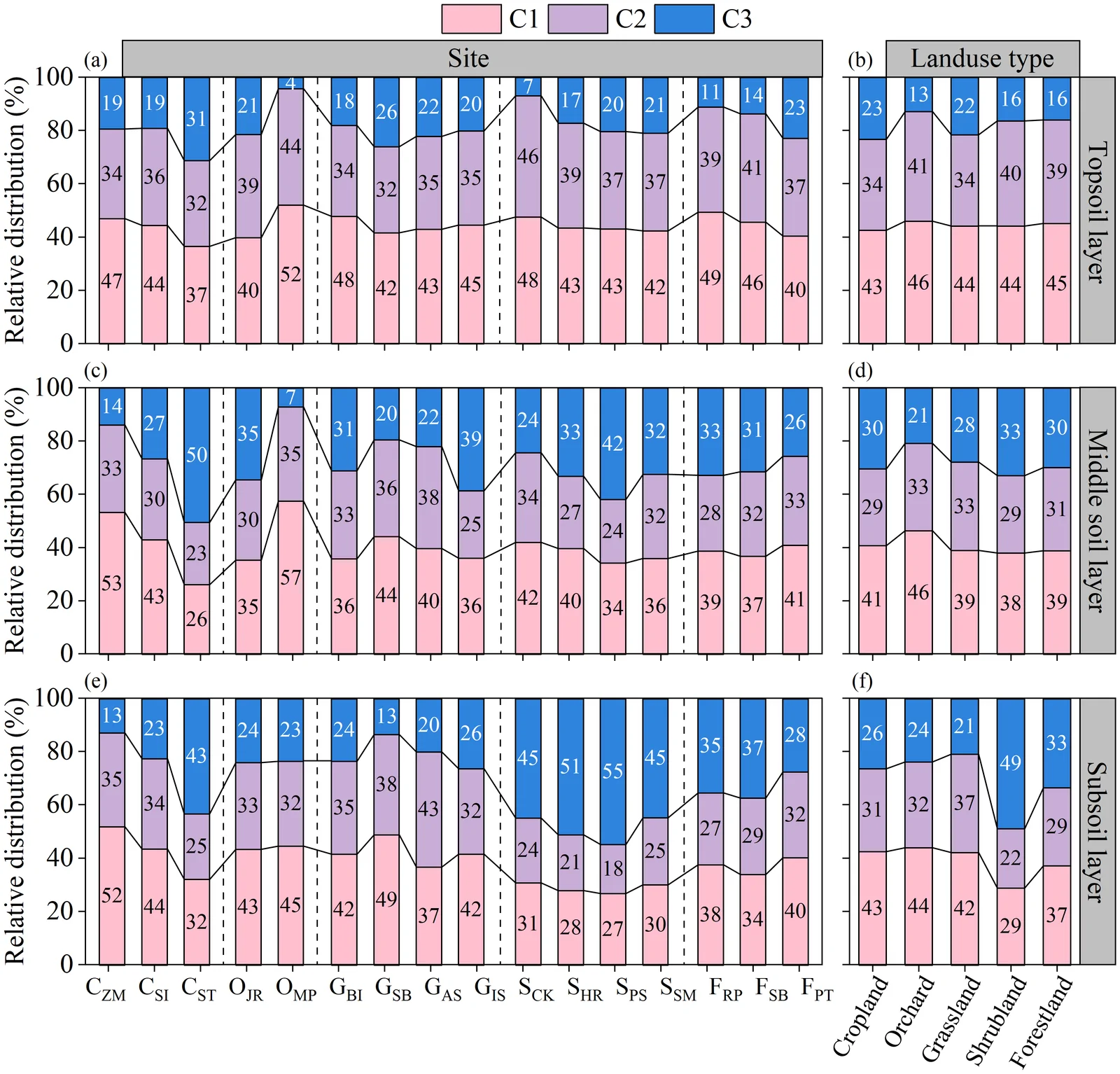
Relative fluorescence contributions (%) of C1, C2, and C3 by site and land use type. Panels **(a, c, e)** show the 16 sites in the topsoil (0–20 cm), middle soil layer (30–50 cm), and subsoil (60–80 cm), respectively; panels **(b, d, f)** show the corresponding land-use means. C1 and C2 are humic-like components, and C3 is protein-like. Site codes are defined in **Table 1**.

In the topsoil, humic-like components (C1 + C2) accounted for more than 70% of total fluorescence across all sites, whereas the protein-like component (C3) contributed a smaller proportion. Cropland and grassland generally showed higher C3 proportions than shrubland, forestland, and orchards, with orchards exhibiting the lowest C3 contribution. In addition, high-input orchards showed a higher humic-like proportion than low-input orchards, whereas root type and restoration mode had little effect on component proportions (**Figures 4a, b**).

In deeper layers, humic-like components still accounted for more than 50% of total fluorescence, but the protein-like fraction increased, especially in shrubland and cropland. Shrubland showed the highest C3 proportion, whereas orchards remained lowest; within cropland, potato sites had an approximately twofold higher C3 proportion than millet and a 3.5-fold higher proportion than maize. In the subsoil, the shrubland C3 proportion further increased to 49%, whereas the effect of orchard management became negligible (**Figures 4c-f**).

**Figure 5:**
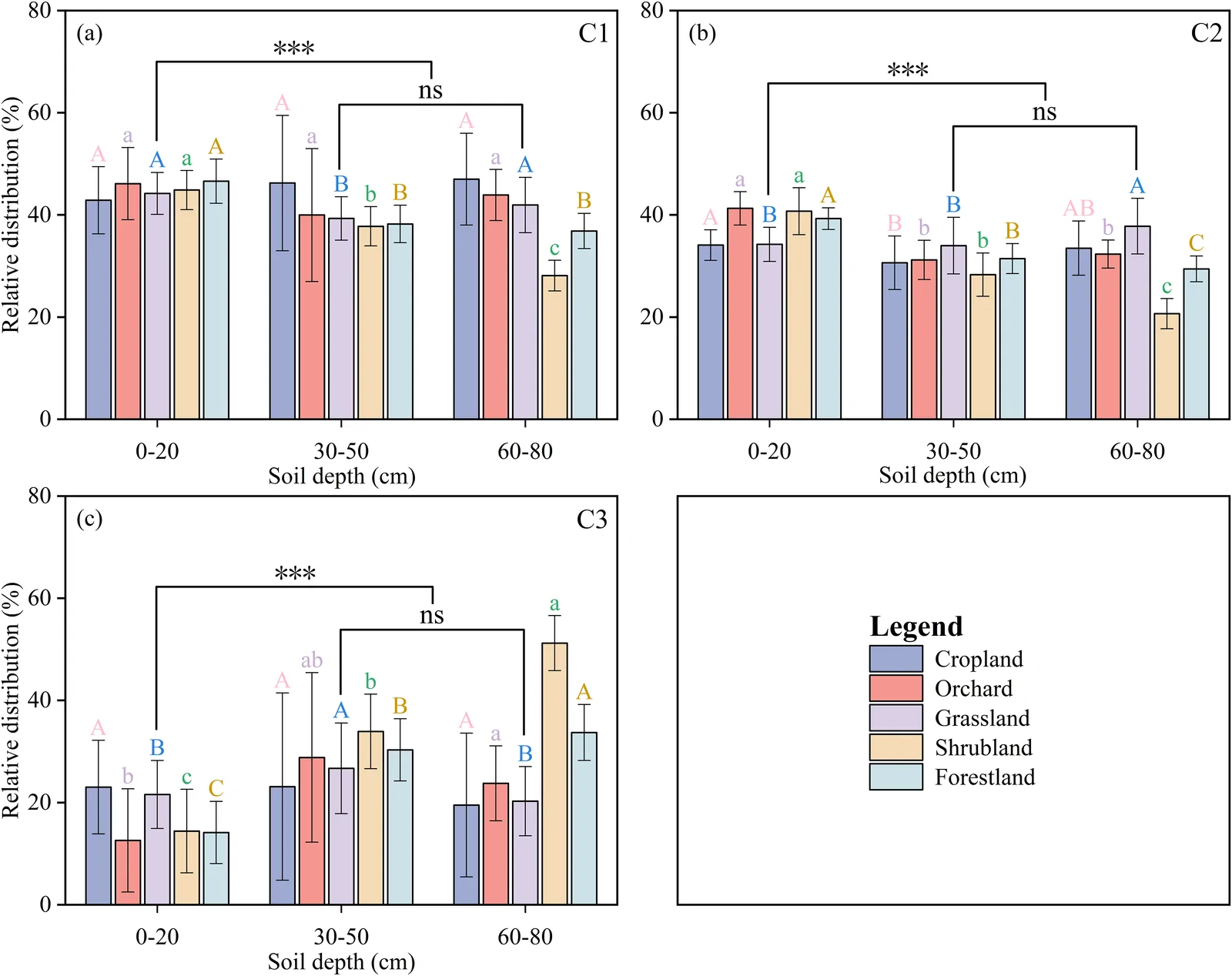
Differences in the relative contributions of DOM fluorescence components among soil layers within each land use type. Panels **(a)**, **(b)**, and **(c)** show components C1, C2, and C3, respectively. Error bars denote SD. Identical letters indicate no significant difference among depths within a land use type (*P* ≥ 0.05), whereas different letters indicate P < 0.05. *** *P* < 0.001; ns, *P* ≥ 0.05.

Vertical differences in component proportions were weak in cropland, where only C2 was higher in the topsoil than in the middle layer (*P* < 0.05). Orchards showed higher topsoil C2 but higher subsoil C3, whereas shrubland and forestland exhibited a clear decrease in C1 and C2 and a corresponding increase in C3 with depth. Overall, C1 and C2 were significantly enriched in topsoil, whereas C3 increased toward deeper layers (*P* < 0.001; **Figures 5a-c**).

### 3.3 Excitation emission matrix fluorescence indices of soil DOM

#### 3.3.1 Variation in FI and BIX of soil DOM

**Figure 6:**
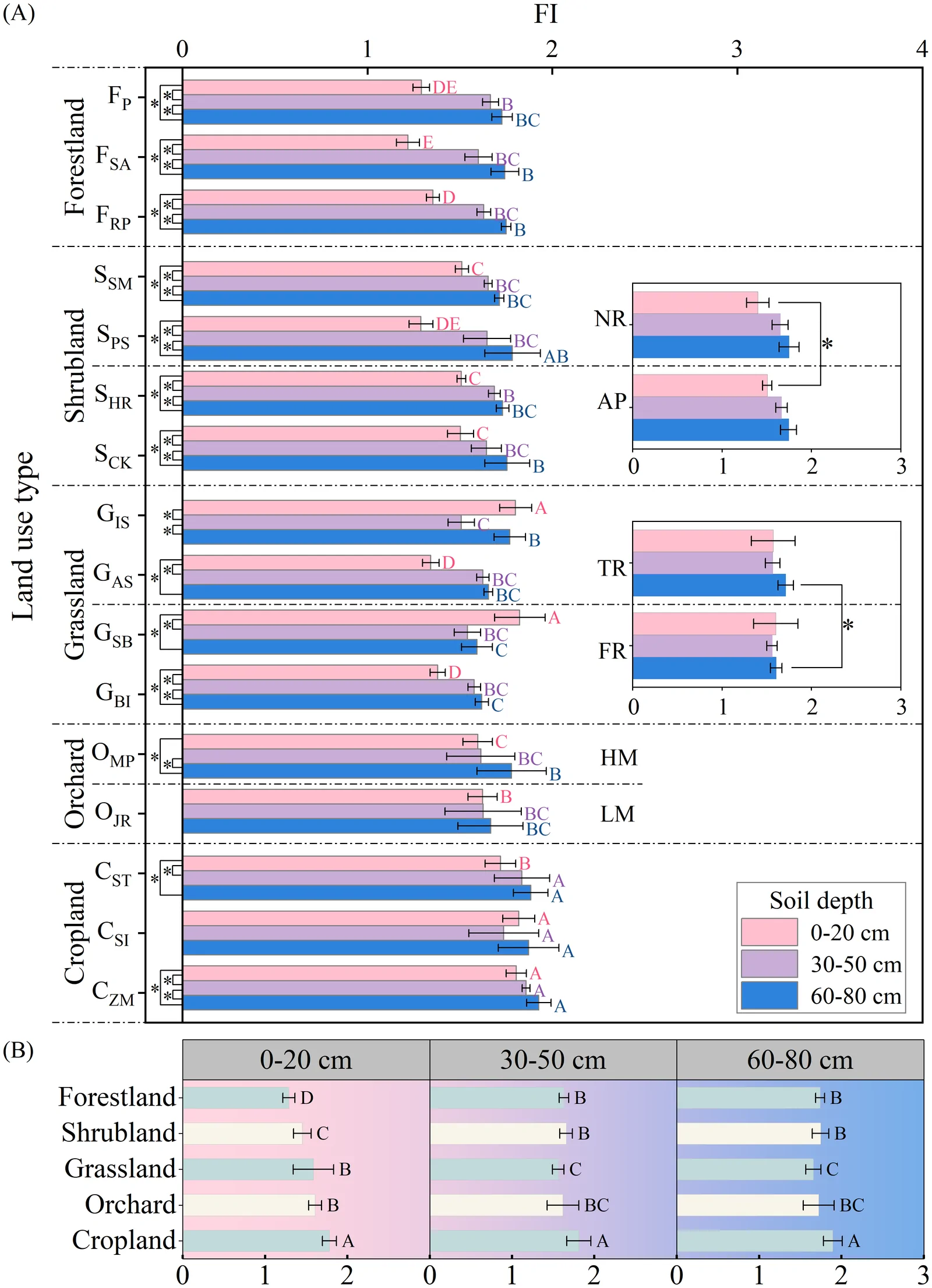
Fluorescence index (FI) values at **(a)** the 16 sampling sites, including within-land-use category summaries, and **(b)** the five land use types at each depth. Pink, purple, and blue bars represent 0-20, 30-50, and 60-80 cm, respectively; error bars denote SD. NR, natural restoration; AP, artificially planted; TR, taproot; FR, fibrous-root; HM, high-input management; LM, low-input management. In panel **(a)**, asterisks beside depth brackets on the left indicate significant differences among depths, and asterisks beside paired-category brackets on the right indicate significant within-land-use contrasts (P < 0.05). Within a depth, bars sharing an uppercase letter are not significantly different among sites in panel **(a)** or among land use types in panel **(b)** (P ≥ 0.05). Site codes are defined in **Table 1**.

**Figure 7:**
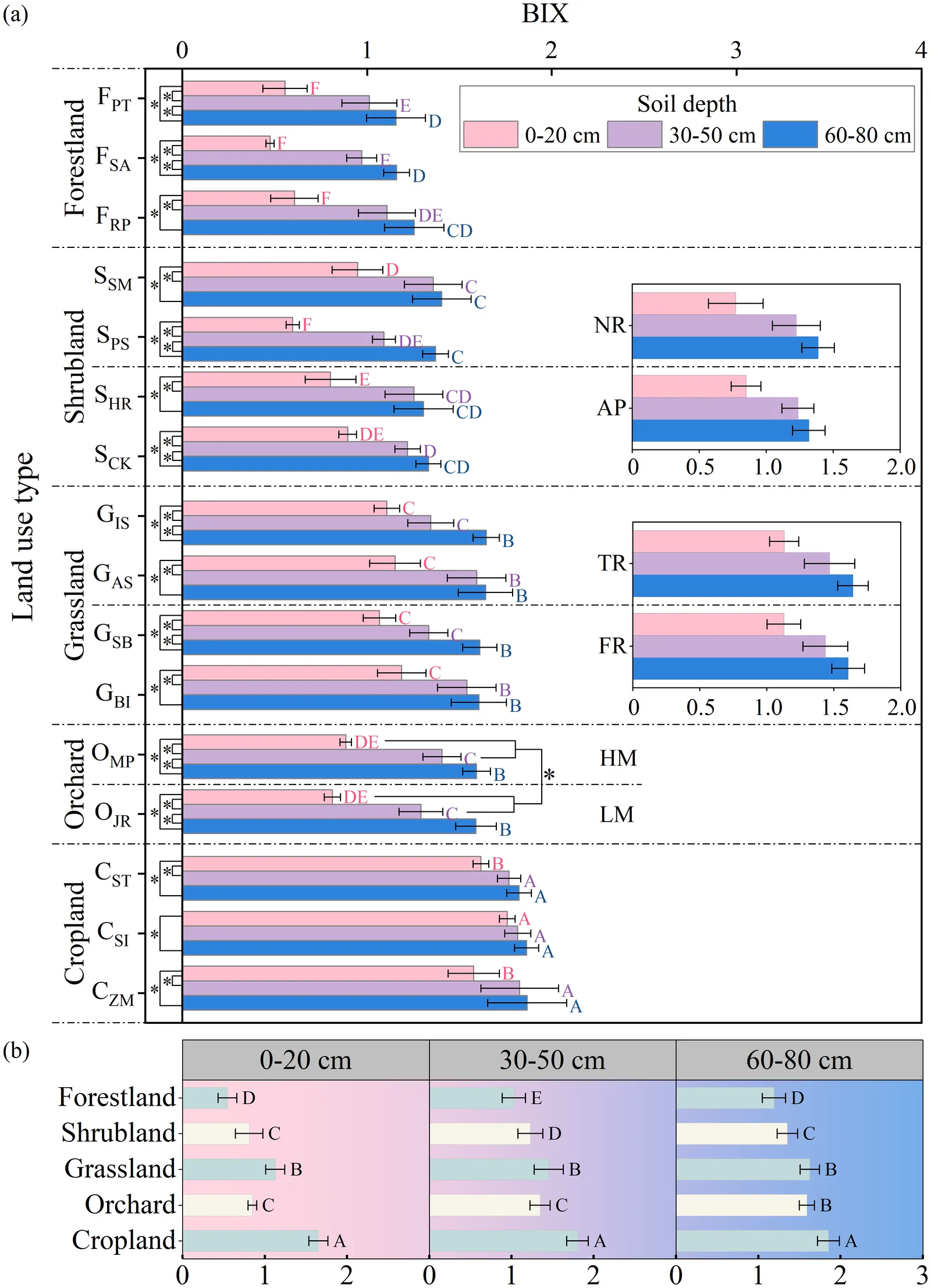
Biological index (BIX) values at **(a)** the 16 sampling sites, including within-land-use category summaries, and **(b)** the five land use types at each depth. Pink, purple, and blue bars represent 0-20, 30-50, and 60-80 cm, respectively; error bars denote SD. NR, natural restoration; AP, artificially planted; TR, taproot; FR, fibrous-root; HM, high-input management; LM, low-input management. In panel **(a)**, asterisks beside depth brackets on the left indicate significant differences among depths, and asterisks beside paired-category brackets on the right indicate significant within-land-use contrasts (P < 0.05). Within a depth, bars sharing an uppercase letter are not significantly different among sites in panel **(a)** or among land use types in panel **(b)** (P ≥ 0.05). Site codes are defined in **Table 1**.

FI and BIX showed similar land-use and depth patterns, although FI differences were less pronounced. Cropland had the highest values, followed by grassland and orchard, whereas shrubland and forestland had the lowest. Both indices generally increased with depth, with topsoil values lower than those in the middle and subsoil layers (P < 0.05; **Figures 6a**, **7a**).

Depth-specific contrasts among land use types were evident for FI and BIX values, particularly for BIX values. In cropland, subsoil BIX values reached 1.85 ± 0.13, decreasing to 1.80 ± 0.13 and 1.65 ± 0.12 in the middle and topsoil layers, respectively. Grassland and orchard exhibited the next highest BIX values, reaching 1.59–1.63 in the subsoil, whereas BIX values in the topsoil and middle soil layer generally did not exceed 1.45. By contrast, shrubland and forestland were characterized by low BIX values, with subsoil BIX values of only 1.19–1.35 and topsoil typically below 0.8. Across all land use types, topsoil BIX values were significantly lower than those in the middle and subsoil layers (*P* < 0.05) (**Figure 7b**). Moreover, across soil layers, FI and BIX values generally followed the order of cropland > grassland > orchard > shrubland > forestland (**Figures 6b**, **7b**).

Within a given land use type, FI and BIX values also varied among vegetation types. In orchards, BIX values under high-input management were significantly higher than under low-input management (*P* < 0.05). In grassland, FI values were significantly higher in taproot sites than in fibrous-root sites (*P* < 0.05). In shrubland, FI values were significantly higher in artificially restored sites than in naturally successional sites (**Figures 6a**, **7a**).

#### 3.3.2 Variation in HIX of soil DOM

**Figure 8:**
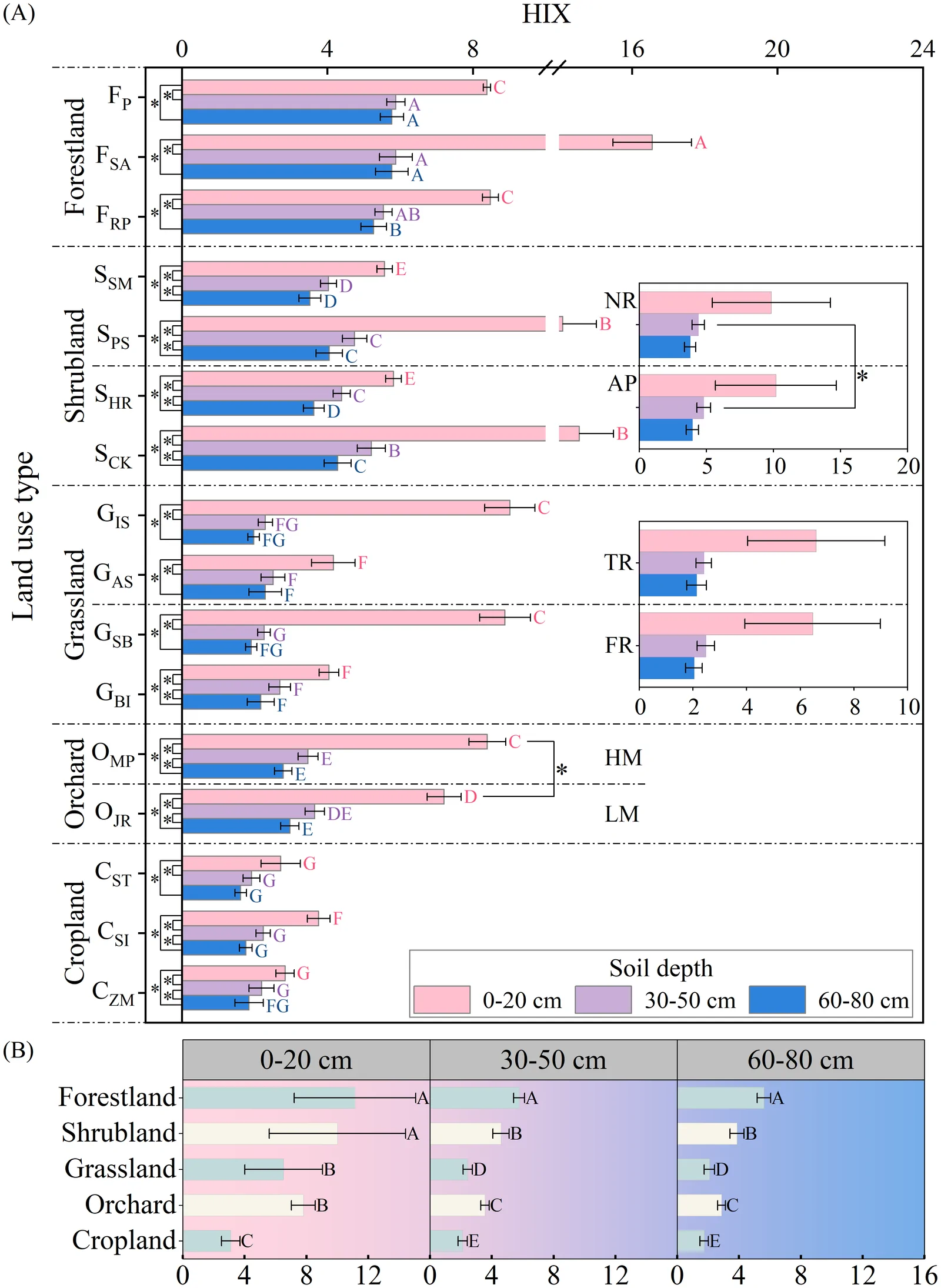
Humification index (HIX) values at **(a)** the 16 sampling sites, including within-land-use category summaries, and **(b)** the five land use types at each depth. Pink, purple, and blue bars represent 0-20, 30-50, and 60-80 cm, respectively; error bars denote SD. NR, natural restoration; AP, artificially planted; TR, taproot; FR, fibrous-root; HM, high-input management; LM, low-input management. In panel **(a)**, asterisks beside depth brackets on the left indicate significant differences among depths, and asterisks beside paired-category brackets on the right indicate significant within-land-use contrasts (P < 0.05). Within a depth, bars sharing an uppercase letter are not significantly different among sites in panel **(a)** or among land use types in panel **(b)** (P ≥ 0.05). Site codes are defined in **Table 1**.

HIX values differed markedly among land use types and soil depths. Overall, forestland exhibited the highest HIX values, followed by shrubland and orchard, whereas grassland and cropland had the lowest HIX values. Along the soil profile, HIX values declined with depth across all land use types, with significantly higher HIX values in the topsoil than in the middle and subsoil layers (*P* < 0.05; **Figure 8a**). Moreover, within each land use type, HIX varied among vegetation types with contrasting traits and management regimes (**Figure 8a**).

The depth-specific differences among land use types were pronounced. In forestland, HIX values in the topsoil reached 11.14 ± 3.93 and decreased to 5.77 ± 0.36 and 5.61 ± 0.44 in the middle and subsoil layers, respectively. In shrubland, HIX values decreased from 10.01 ± 4.41 in the topsoil to 4.59 ± 0.53 and 3.87 ± 0.45 in the middle and subsoil layers. In orchards, topsoil HIX values reached 7.80 ± 0.77, whereas middle- and subsoil remained below 3.5. Grassland and cropland exhibited consistently low HIX values, with topsoil values ranging from 3.10 to 6.53 and subsoil values generally below 2.1. Across land use types, topsoil HIX values were significantly higher than those in the middle and subsoil layers (*P* < 0.05), whereas differences between the middle and subsoil layers were significant only at some sites (**Figure 8b**). Furthermore, across soil layers, HIX generally followed the order of forestland > shrubland > orchard > grassland > cropland. However, differences between forestland and shrubland, and between orchard and grassland, were not significant in the topsoil but became significant in the middle and subsoil layers (*P* < 0.05; **Figure 8b**).

Within land use types, vegetation-related contrasts in HIX values were most evident in the topsoil. In forestland, topsoil HIX values ranged from 8.39 to 15.56 across tree species, while the ranges were much narrower in the middle (5.54–5.88) and subsoil layers (5.27–5.77). Willow exhibited a significantly higher topsoil HIX than other tree species (*P* < 0.05), but this difference diminished with increasing depth. In orchards, topsoil HIX values differed significantly between management regimes, with higher values under low-input management than under high-input management (*P* < 0.05). Notably, shrubland and grassland also showed significant vegetation-type differences in topsoil HIX values, but these differences were not associated with root type or restoration mode. In shrubland, S_CK_ and S_PS_ exhibited significantly higher HIX values than the other shrub species in the topsoil (*P* < 0.05), while in grassland, G_SB_ and G_IS_ had higher HIX values than the other grassland types in the topsoil (*P* < 0.05). By contrast, cropland showed no clear differences among crops across depths. Overall, vegetation-type effects within a land use category were strongest in the topsoil and weakened with depth (**Figure 8a**).

### 3.4 UV–visible spectral indices of soil DOM

**Figure 9:**
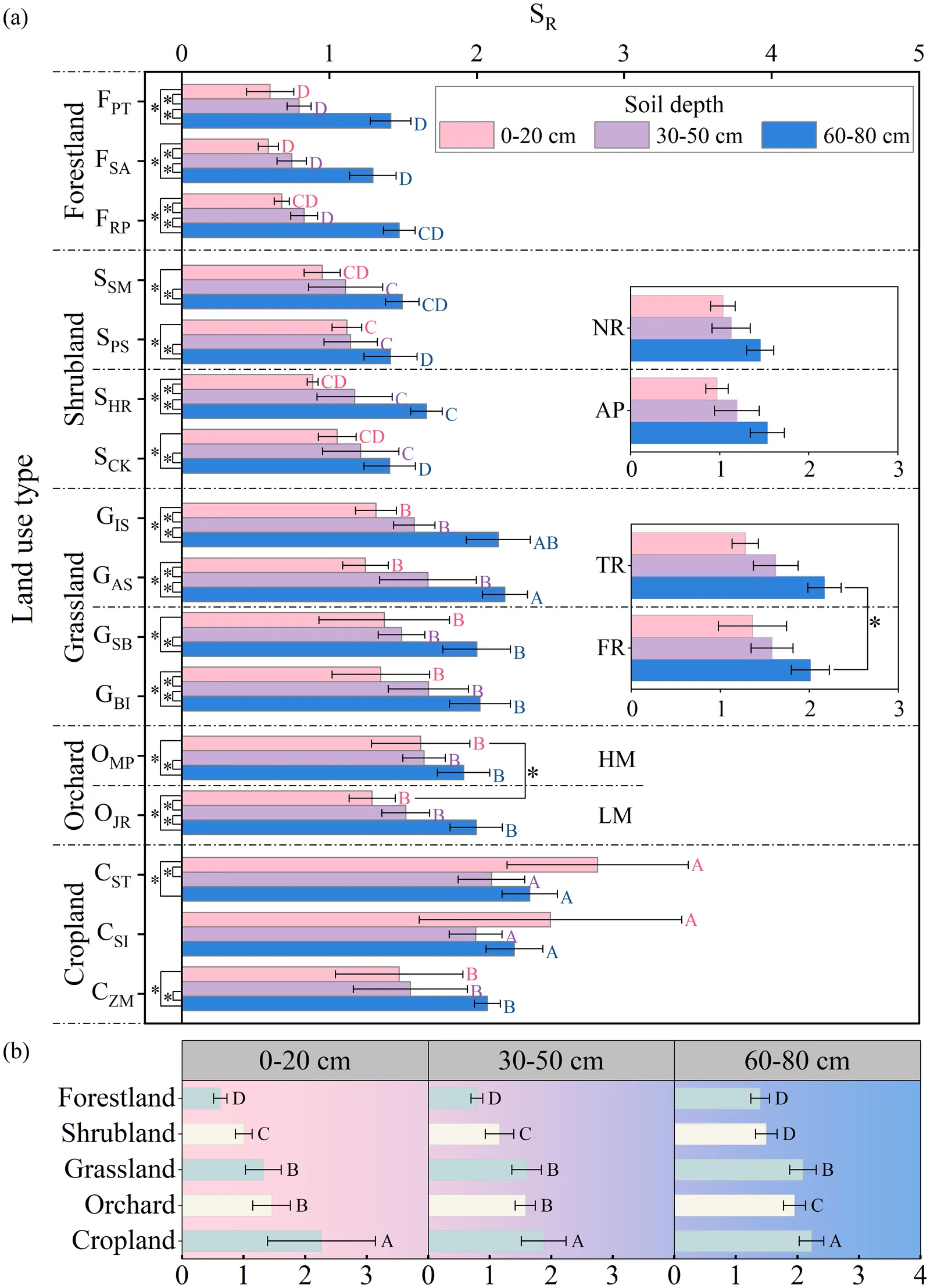
Spectral slope ratio (SR) values at **(a)** the 16 sampling sites, including within-land-use category summaries, and **(b)** the five land use types at each depth. Pink, purple, and blue bars represent 0-20, 30-50, and 60-80 cm, respectively; error bars denote SD. NR, natural restoration; AP, artificially planted; TR, taproot; FR, fibrous-root; HM, high-input management; LM, low-input management. In panel **(a)**, asterisks beside depth brackets on the left indicate significant differences among depths, and asterisks beside paired-category brackets on the right indicate significant within-land-use contrasts (P < 0.05). Within a depth, bars sharing an uppercase letter are not significantly different among sites in panel **(a)** or among land use types in panel **(b)** (P ≥ 0.05). Site codes are defined in **Table 1**.

**Figure 10:**
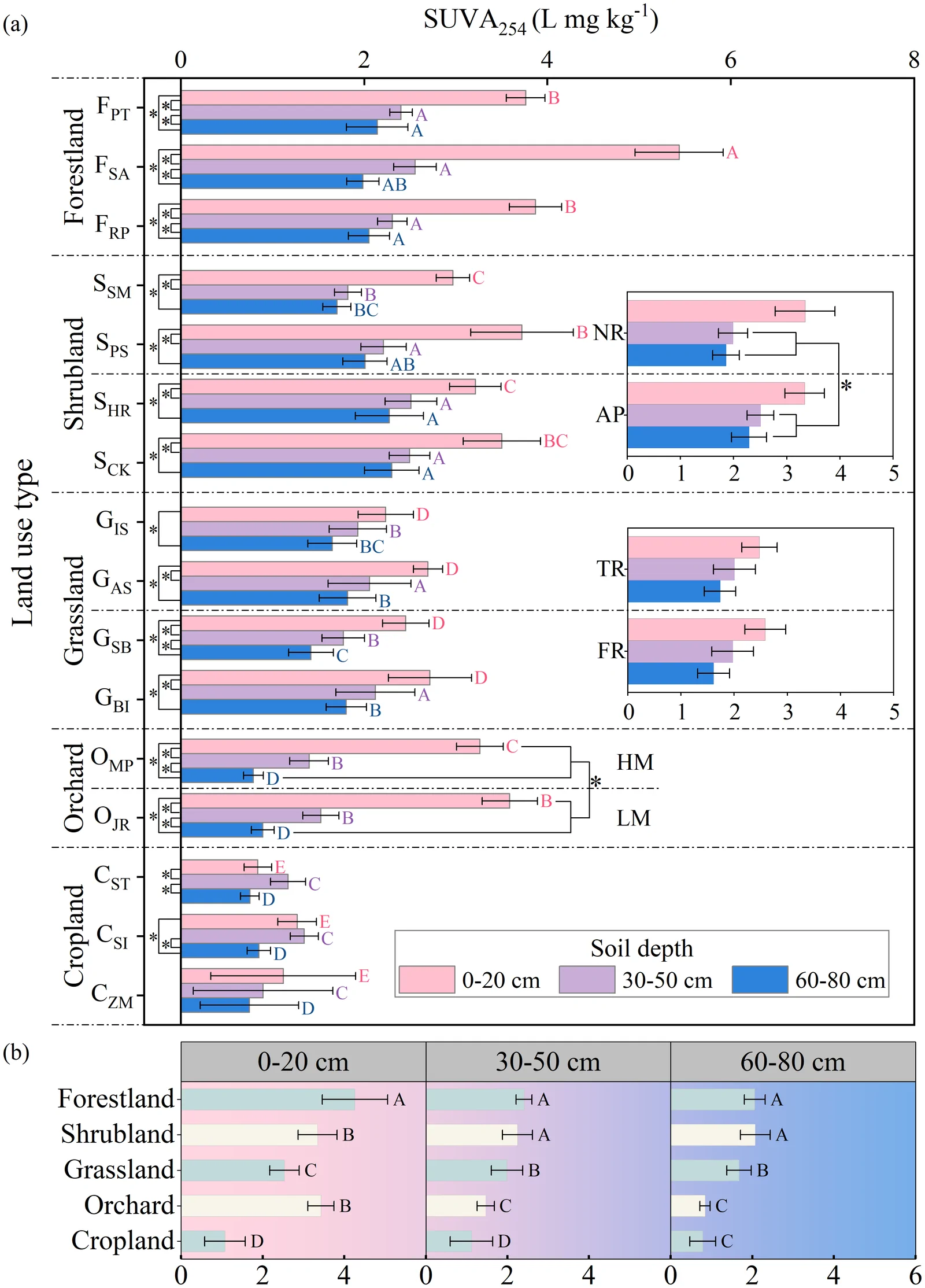
Specific UV absorbance at 254 nm (SUVA254) values at **(a)** the 16 sampling sites, including within-land-use category summaries, and **(b)** the five land use types at each depth. Pink, purple, and blue bars represent 0-20, 30-50, and 60-80 cm, respectively; error bars denote SD. NR, natural restoration; AP, artificially planted; TR, taproot; FR, fibrous-root; HM, high-input management; LM, low-input management. In panel **(a)**, asterisks beside depth brackets on the left indicate significant differences among depths, and asterisks beside paired-category brackets on the right indicate significant within-land-use contrasts (P < 0.05). Within a depth, bars sharing an uppercase letter are not significantly different among sites in panel **(a)** or among land use types in panel **(b)** (P ≥ 0.05). Site codes are defined in **Table 1**.

The S_R_ and SUVA_254_ values differed significantly among land use types and across soil depths, and the two indices showed opposite trends. Overall, forestland exhibited the highest SUVA_254_ values and the lowest S_R_ values, followed by shrubland and grassland, whereas orchard and cropland showed the lowest SUVA_254_ values and the highest S_R_ values. Along the soil profile, S_R_ generally increased with depth whereas SUVA_254_ decreased with depth across land use types (**Figures 9a**, **10a**). Meanwhile, within each land use type, S_R_ and SUVA_254_ values varied among vegetation types with contrasting traits and management regimes (**Figures 9a**, **10a**).

Depth-specific contrasts among land use types were clear. In cropland, topsoil S_R_ values reached 2.27 ± 0.88, decreasing to 1.88 ± 0.36 in the middle soil layer and increasing again to 2.23 ± 0.20 in the subsoil; meanwhile, SUVA_254_ values remained below 1.1 across all depths. In orchards and grassland, subsoil S_R_ values ranged from 1.95 to 2.1, whereas S_R_ in the topsoil and middle soil layer declined to 1.3–1.6; SUVA_254_ values reached 2.5–3.4 in the topsoil and remained below 2 in the middle and subsoil layers. In shrubland and forestland, S_R_ values were consistently <1.5 across depths, whereas SUVA_254_ reached 3.4–4.3 in the topsoil and remained ≥2 in deeper layers. Except for cropland, subsoil S_R_ values were significantly higher than those in the topsoil and middle soil layers (*P* < 0.05), while SUVA_254_ values were significantly higher in the topsoil than in the middle and subsoil layers (**Figures 9b**, **10b**). Furthermore, across soil layers, S_R_ values generally followed the order of cropland > orchard and grassland > shrubland > forestland. In the middle and subsoil layers, SUVA_254_ values generally followed forestland and shrubland > grassland > orchard and cropland. In addition, differences among land use types became less pronounced with increasing depth (**Figures 9b**, **10b**).

Within a given land use type, S_R_ and SUVA_254_ also varied among vegetation types and management or restoration regimes. In forestland, willow exhibited significantly higher SUVA_254_ values (5.44) than the other two tree types (*P* < 0.05). In orchards, low-input management was associated with lower SR in the topsoil but higher SUVA254 in both the topsoil and subsoil compared with high-input management (*P* < 0.05). In grassland, subsoil S_R_ was significantly higher in taproot sites than in fibrous-root sites (*P* < 0.05). In shrubland, SUVA_254_ in the middle and subsoil layers was significantly higher under artificially restored sites than in naturally successional sites (*P* < 0.05). In cropland, maize and the other two crops differed in their vertical patterns. Maize exhibited the lowest S_R_ values and the highest SUVA_254_ values in the topsoil, whereas this feature occurred in the middle soil layer for the other crops (**Figures 9a**, **10a**).

### 3.5 Principal component analysis and correlation analysis of soil DOM

**Figure 11:**
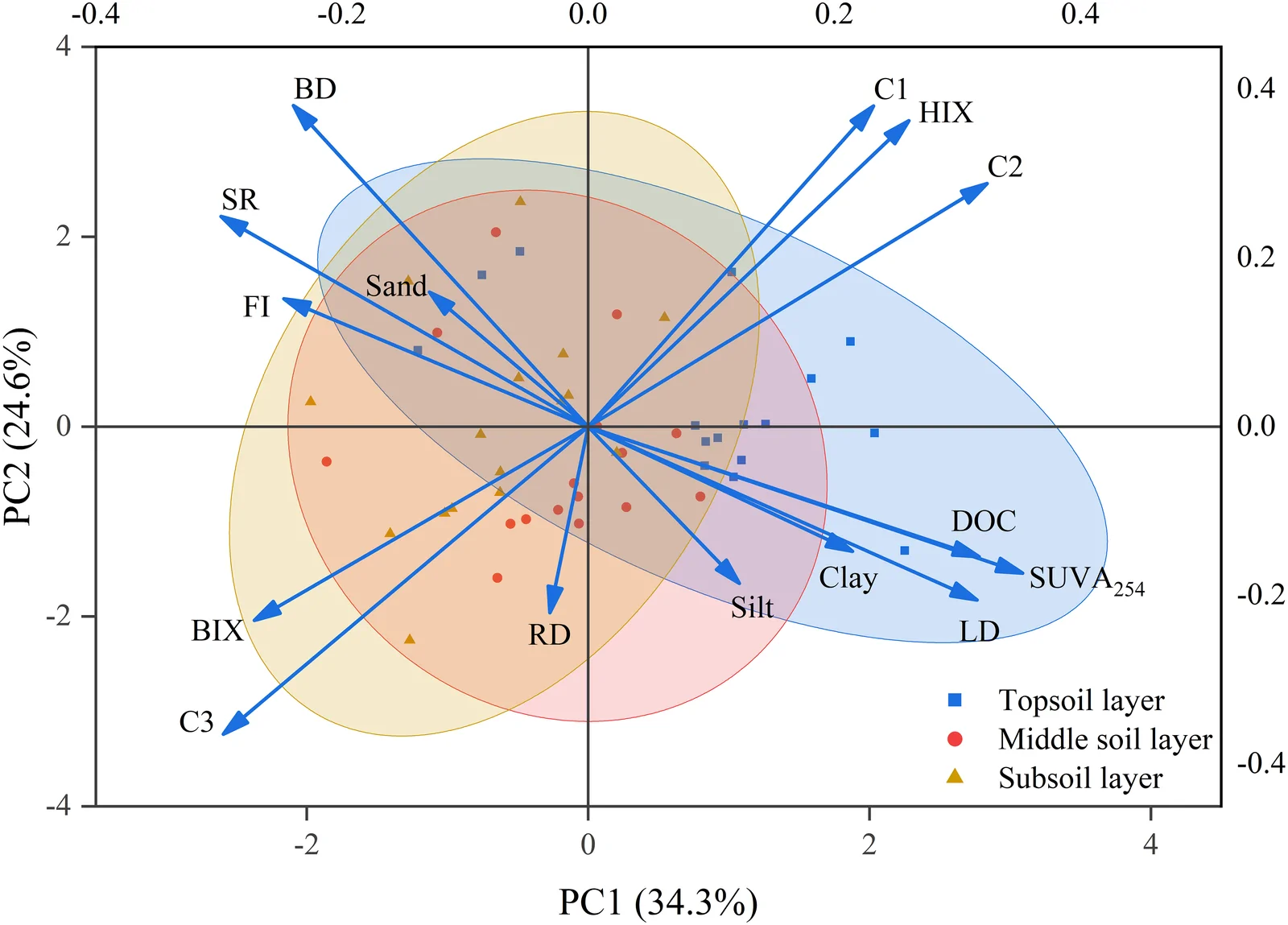
Principal component analysis (PCA) biplot of soil DOM optical parameters, fluorescence-component proportions, root density (RD), litter density (LD), bulk density (BD), and soil texture. Symbols represent site-depth means (n = 48): blue squares, red circles, and ochre triangles denote topsoil, middle soil, and subsoil observations, respectively. Colored ellipses delineate the three depth groups, and vectors show variable loadings.

PCA was used to visualize multivariate variation among the 48 site-depth observations in relation to land use type and soil depth. The first two principal components (PC1 and PC2) explained 34.3% and 24.6% of the total variance, respectively. The three depth groups overlapped but showed a gradient along PC1, with topsoil observations tending toward positive scores and middle- and subsoil observations shifting toward negative scores. PC1 was positively correlated with DOC, SUVA_254_, HIX, components C1 and C2, LD, and the fine-texture fractions (clay and silt), but negatively correlated with FI, BIX, S_R_, BD, RD, component C3, and sand content. Vector geometry further indicated small angles between LD, SUVA_254_, DOC, and clay, as well as between BD, S_R_, FI, and sand. In addition, C1 and C2 were positioned close to HIX, whereas C3 was positioned close to BIX (**Figure 11**).

**Figure 12:**
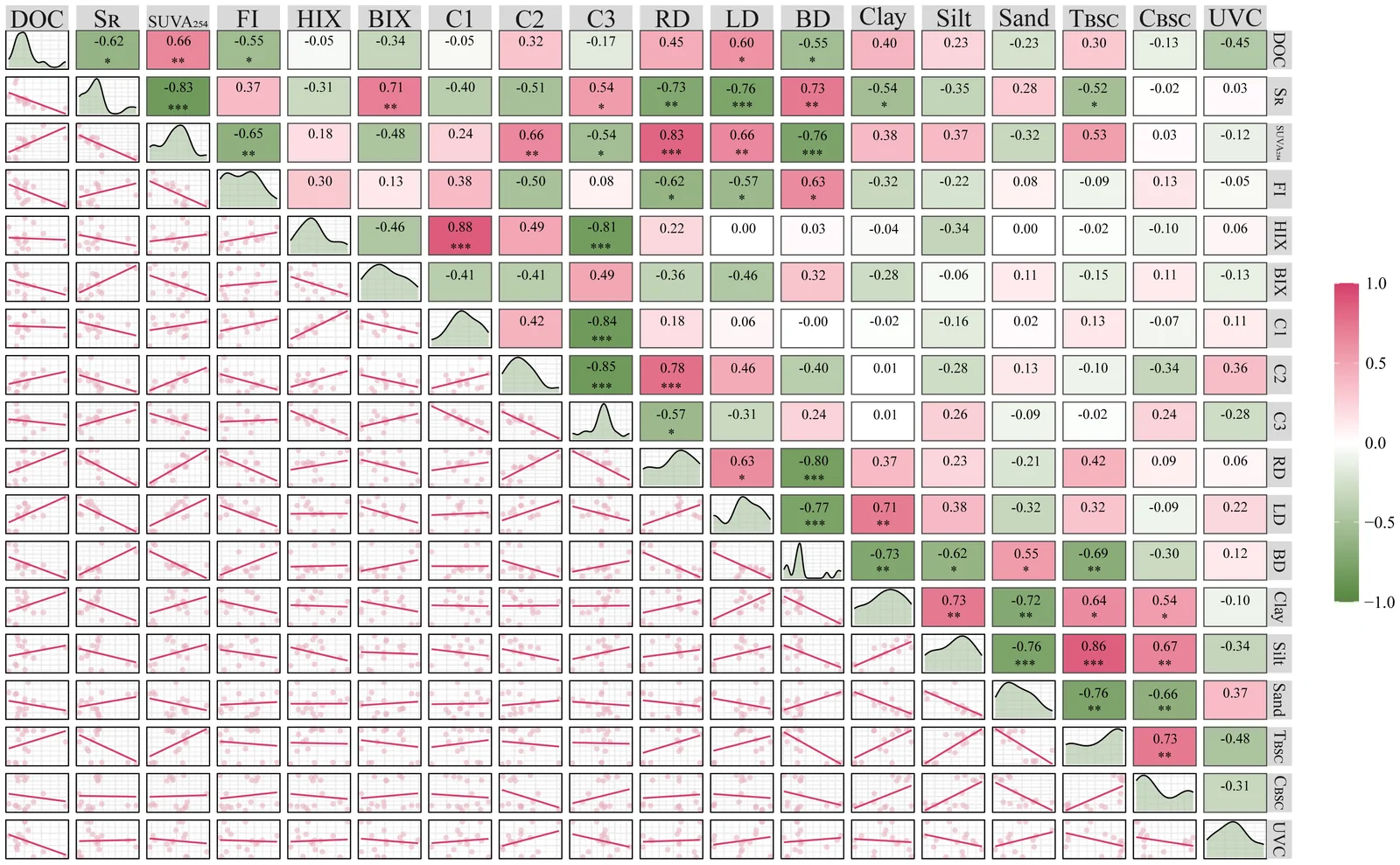
Pearson correlation matrix among soil DOM optical parameters, fluorescence-component proportions, RD, LD, BD, soil texture, biological soil crust thickness (T_BSC_) and coverage (C_BSC_), and understory vegetation coverage (UVC) in topsoil. Asterisks indicate significance levels of Pearson correlation coefficients: *P < 0.05, **P < 0.01, and ***P < 0.001.

Pearson correlation analysis (**Figure 12**) further supported these patterns for the topsoil. S_R_ was strongly positively correlated with BIX and BD (*P* < 0.01), but negatively correlated with SUVA_254_, RD, and LD (*P* < 0.01). SUVA_254_ was strongly positively correlated with DOC, C2, RD, and LD (*P* < 0.01), and negatively correlated with S_R_, FI, and BD (*P* < 0.01); FI showed correlation patterns similar to S_R_. HIX was strongly positively correlated with C1 and negatively correlated with C3 (*P* < 0.01). BD was strongly negatively correlated with RD, LD, T_BSC_, and clay content (*P* < 0.01). For surface properties, T_BSC_ was strongly positively correlated with silt content and C_BSC_, but negatively correlated with BD and sand content (*P* < 0.01, **Figure 12**).

## 4 Discussion

### 4.1 Spatial variation in soil DOC content

In this study, differences in DOC content among land use types were most evident in the topsoil (**Figure 2**), with higher values in forestland and shrubland, followed by grassland, and lower values in orchards and cropland. This pattern was associated mainly with differences in surface organic inputs and near-surface processes (Chantigny, 2003; Sun et al., 2026).

At the land use scale, differences in DOC content can be explained by three aspects, which include organic inputs, soil microenvironment, and physical retention (Han et al., 2024; Zhang et al., 2025). First, variation in organic inputs was strongly associated with topsoil DOC. Forestland had the highest DOC content, consistent with its higher litter density (LD), root density (RD), and biological soil crust thickness (T_BSC_) (**Supplementary Figure 1**). In contrast, cropland had relatively low LD and RD across depths (**Supplementary Figures 1a-c**). PCA showed a positive association between DOC and litter density, consistent with an important role of surface organic inputs in DOC variability (**Figure 11**). Second, land use influenced DOC retention by altering soil texture and structure. Cropland had a lower clay content but a higher sand content (**Supplementary Figures 1a-c**), which may promote DOC loss via infiltration and leaching and reduced retention, consistent with the mechanism proposed by Kaiser and Kalbitz (2012). Third, land use effects on soil temperature and moisture were not measured here, but published studies indicate that these conditions can influence DOC production and transport (He et al., 2024; Young et al., 2021). For example, higher water-holding capacity in grassland and deeper soil drying in forestland in arid and semiarid regions may alter DOC transport along the profile, although this effect mainly modified vertical patterns rather than driving overall land use differences (He et al., 2024; van Dijk and Keenan, 2007).

DOC content decreased significantly with increasing soil depth across all land use types (*P* < 0.05), although the magnitude of decline varied. This depth-related decline is consistent with the surface enrichment of LD and the progressive weakening of fresh organic inputs with depth (**Supplementary Figure 1**). In deeper layers, DOM is sustained mainly by limited deep-root inputs or the vertical transport of DOM from the surface, while mineral sorption and continuous microbial consumption further reduce the DOC pool (Kaiser and Kalbitz, 2012; Teixeira et al., 2024). Importantly, the decline rate varied among land use types. In the topsoil, orchards, grassland, and shrubland had relatively high surface inputs, which partly explains why their DOC content could overlap in the topsoil. In the middle and subsoil layers, however, shrubland maintained higher DOC content than grassland and orchards (**Figure 2b**), likely due to deeper carbon inputs from woody roots. Forestland showed the steepest decline with depth, consistent with strong surface litter and root inputs but a rapid reduction in carbon supply and microbial activity with depth (Wang et al., 2024). Grassland exhibited a more gradual decrease, consistent with relatively high root density and well-developed biological soil crusts that support DOC turnover and retention (**Supplementary Figure 1**) (Riveras-Muñoz et al., 2022; Szyja et al., 2023).

Within land use types, management and vegetation types further modified DOC content. In orchards, the combined species-management contrast was associated with DOC content, with consistently lower values at the apple/high-input site than at the walnut/low-input site across depths (*P* < 0.05; **Figure 2b**). The observed difference may reflect greater surface cover and carbon retention at the walnut/low-input site (Wang et al., 2024) and stronger disturbance at the apple/high-input site, but species and management effects could not be separated in this field comparison. In grassland, contrasting root types can alter root distribution and rhizosphere inputs (Peixoto et al., 2020; Teixeira et al., 2024), yet DOC profiles did not differ clearly between root systems. This suggests that root-derived inputs may be reshaped by subsequent leaching, microbial processing, and mineral sorption (Leinemann et al., 2018; Nakhavali et al., 2020). In shrubland, artificially planted sites (AP) showed higher RD and higher understory vegetation cover than naturally restored sites (NR) (*P* < 0.05; **Supplementary Figure 1**), but DOC did not differ significantly between AP and NR. This suggests that greater organic inputs in AP may have been partly offset by faster microbial processing and DOC consumption (Wang et al., 2023a).

### 4.2 Spatial variation in DOM fluorescence components

At the land use scale, fluorescence component patterns reflected how vegetation type and disturbance intensity reshape DOM sources. Across all land use types, humic-like components dominated the total fluorescence signal (**Figure 4**), which was consistent with a substantial contribution from plant-derived material. This agrees with observations from dryland sites in Israel, where DOM was largely controlled by plant residues and vegetation-derived organic matter, with optical indicators pointing to strong plant-derived and humic-like substances (Nasonova et al., 2022; Rinot et al., 2021). Although humic-like components were dominant everywhere, the protein-like fraction tended to be relatively higher in cropland and grassland and to increase strongly in shrubland with depth, while orchards generally exhibited the lowest protein-like contribution (**Figure 4**). Such differences likely reflect contrasts in organic input quality and microbial processing. Forestland and shrubland typically provide lignin- and polyphenol-rich litter and more stable organic inputs, which can be transformed by microbes into larger and more aromatic humic-like DOM, leading to higher humic-like signatures (Chen et al., 2023; Hensgens et al., 2021; Kaiser and Kalbitz, 2012). Supporting this, humic-like components were positively related to RD, and land use types with higher RD tended to show stronger humic-like substances (**Supplementary Figure 1**). This highlights root systems as an important link between land use and DOM composition, because higher RD can enhance rhizosphere carbon inputs and microbial transformation, promoting humic-like DOM formation. Similar patterns have been reported on the Loess Plateau, where forestland DOM showed higher aromaticity and hydrophobicity as well as stronger humic-like features than cropland (Liu et al., 2015).

Vertically, the topsoil was generally enriched in humic-like substances, while the relative contribution of protein-like components increased with depth, suggesting a shift toward more microbially processed or fresh characteristics in deeper DOM (Meng et al., 2024; Wang et al., 2024). This depth trend is consistent with Loess Plateau profile studies showing declining humic-like substances and increasing protein-like substances with profile (Li et al., 2019; Meilleur et al., 2023). Except for cropland, the protein-like component was relatively low in the topsoil, which may reflect long-term microbial assimilation of small protein-like molecules and their conversion into larger humic-like substances during sustained revegetation. The strong negative relationships between C3 and both C1 and C2 (*P* < 0.001) further indicate contrasting distribution patterns between protein-like and humic-like components, consistent with differential processing of these fluorophores along the soil profile (Kaiser and Kalbitz, 2012; Ren and Cai, 2025). In addition, stronger litter inputs in the topsoil (**Supplementary Figure 1**) promote humic-like formation and stabilization through mineral adsorption and complexation, resulting in significantly higher C1 and C2 contributions in the topsoil than in deeper layers (*P* < 0.05) (Meilleur et al., 2023; Williams et al., 2013). With increasing depth, decreasing C1 and C2 contributions suggest that deep DOM is not a simple extension of surface DOM into deeper layers. Instead, deep root activity and microbial metabolism under substrate limitation may generate more microbially derived soluble products, increasing the relative protein-like signal (Han et al., 2023; Lavallee et al., 2020).

Within land use types, management and restoration factors acted mainly as modifiers of component proportions. In orchards, high-input management at the apple site involved more frequent disturbance and reduced litter return, which may have contributed to lower protein-like fluorescence than at the walnut/low-input site; because tree species and management co-varied, their effects could not be separated. In shrubland, artificially planted sites often had higher understory coverage and higher LD, providing more terrestrial organic inputs and thus a higher humic-like component in the topsoil. With increasing depth, differences in surface inputs weakened, and the component proportions of AP and NR tended to converge (**Figure 4**). This pattern is consistent with the idea that deep DOM is mainly controlled by transport, mineral interactions, and limited inputs within the subsoil (Charamba et al., 2025; Fröberg et al., 2007).

### 4.3 Spatial variation in fluorescence spectral indices of soil DOM

Fluorescence indices provided optical evidence related to DOM processing and humification (Huguet et al., 2009; Ohno, 2002). FI varied little among land use types (**Figure 6**), whereas BIX and HIX showed clearer contrasts. Higher BIX in cropland and grassland was consistent with stronger microbially associated or freshly produced DOM signatures, whereas higher HIX and lower BIX in forestland and shrubland were consistent with more humified and plant-associated optical signatures (Cory and McKnight, 2005; Kaiser and Kalbitz, 2012). FI, BIX, and fluorescence-component assignments are proxies influenced by source, processing history, and overlapping fluorophores and therefore do not uniquely identify DOM origin.

These patterns can be linked to disturbance intensity and substrate quality. In cropland, frequent disturbance was associated with higher bulk density (BD) and lower LD and RD (**Supplementary Figure 1**), favoring rapid microbial cycling of labile substrates. Under such conditions, microbial metabolites can contribute a larger fraction of DOM, which could contribute to higher FI and BIX and lower HIX. In forestland and shrubland, weaker disturbance and larger inputs of woody litter may promote repeated processing and accumulation of humic-like substances, resulting in higher HIX and lower FI and BIX (Zethof et al., 2019). Soil structure and near-surface features can further regulate these indices. In cropland, high BD and a constrained pore structure may promote rapid cycles of DOM production and consumption and thereby strengthen microbial signals. In contrast, in forestland and grassland topsoil, more stable soil structure and stronger BSCs development (**Supplementary Figure 1**) can enhance physical protection of DOM on mineral surfaces or within aggregates, allowing more extensive processing and humification (Chamizo et al., 2017; Liu et al., 2015; Wilson and Xenopoulos, 2009).

A second noteworthy pattern was that significant vegetation-type differences in topsoil indices, especially HIX, appeared in grassland and shrubland; but these differences did not correspond clearly to root type or restoration mode. This suggests that site-to-site factors, such as microbial activity, litter chemistry, or microhabitat conditions, may outweigh the single-factor contrasts defined in our design. Higher microbial activity, for instance, could promote repeated processing and thus enhance humic substances, leading to higher HIX in certain vegetation types (Zethof et al., 2019).

Across all land use types, DOM generally shifted from a more humified and aromatic state in the topsoil to a deeper DOM pool with stronger microbially associated optical signatures and smaller apparent molecular size. This vertical differentiation does not mean that deep DOM is simpler, but rather it reflects stronger environmental constraints on DOM formation in deeper horizons. Compared with topsoil, the source structure of deep DOM likely depended more on vertical transport of soluble carbon and deep-root inputs, rather than on direct inputs of plant residues (Beillouin et al., 2023; Szyja et al., 2023). The PCA also supports this interpretation (**Figure 11**). Mechanistically, topsoil receives concentrated litter and rhizosphere inputs and may experience more favorable moisture or temperature conditions for repeated decomposition and re-synthesis, promoting humic-like DOM and higher HIX. In deeper layers, limited carbon and energy supply, together with selective retention of large and aromatic DOM during transport, can increase the relative intensity of microbially associated fluorescence and raise FI and BIX while lowering HIX (Fan et al., 2025; Hensgens et al., 2021).

Within land use types, species-management contrasts were associated with the balance between fresh and humified substances, but the effects were often weaker than the main land use contrast. In orchards, the apple/high-input and walnut/low-input sites differed in BIX and HIX. The apple/high-input site showed stronger fresh, microbially associated signatures, whereas the walnut/low-input site showed stronger humification (**Figures 7** and **8**). Intensive orchard management can reduce humic substances by lowering surface organic matter accumulation and increasing disturbance (Knapen et al., 2007; Liu et al., 2015; Messing et al., 2003). However, the observed differences should not be attributed to management alone because tree species, litter chemistry, and management-related changes in soil properties can jointly influence microbial processing and mineral stabilization. In grassland and shrubland, root type and restoration mode showed relatively weak effects on FI, BIX, and HIX. A likely reason is that newly produced DOM can be quickly consumed or stabilized by minerals and aggregates, which can reduce persistent differences in optical indices at the profile scale. Similarly, deeper root inputs may strengthen microbial contribution locally, but these effects may be weakened when DOM is integrated across soil horizons and transport pathways (Rinot et al., 2021; Witzgall et al., 2024).

### 4.4 Spatial variation in UV–Visible spectral indices of soil DOM

Across land use types, S_R_ and SUVA_254_ exhibited opposite patterns, with higher S_R_ but lower SUVA_254_ in cropland and grassland, and the reverse in shrubland and forestland (**Figures 9b**, **10b**). Lower S_R_ in woody systems was consistent with more humified DOM of larger apparent molecular size, whereas higher S_R_ in cropland and grassland was consistent with smaller apparent molecular size and more microbially associated optical signatures. This interpretation is consistent with the component-based results, which show relatively higher protein-like contributions in cropland and grassland, whereas humic-like components dominate in woody systems (**Figure 4**). Woody litter inputs typically contain more lignin and polyphenols; after microbial processing, these inputs more readily form aromatic, humic-like DOM, leading to higher SUVA_254_ and lower S_R_. Conversely, crop residues and root inputs often contain more easily decomposed carbohydrates and lower-aromatic substrates, which may yield optical signatures of lower aromaticity and smaller apparent molecular size. Frequent disturbance may also weaken consistent depth contrasts in cropland (Weishaar et al., 2003; Zethof et al., 2019).

Along soil profiles, S_R_ generally increased and SUVA_254_ generally decreased with depth across land use types. Together with the PCA and correlation results (**Figures 11**, **12**), this pattern indicates that deep DOM differed strongly from topsoil DOM. Because LD was concentrated near the surface (**Supplementary Figure 1**), deep DOM likely depended less on subsoil litter inputs and more on vertical transport from the surface and limited deep-root inputs. During transport, DOM fractions with optical signatures of greater aromaticity and larger apparent molecular size may be preferentially retained through mineral adsorption, leaving deeper DOM with smaller apparent molecular size and weaker aromatic optical signatures (Helms et al., 2008; Kaiser and Kalbitz, 2012; Rinot et al., 2021). However, depth responses were not strictly linear across all land uses. Differences in soil structure stability and root input patterns may change the magnitude of these shifts (Sae-Tun et al., 2023; Witzgall et al., 2024). For example, in forestland and shrubland, deep roots and relatively stable soil structure may partly buffer the decline in aromaticity with depth, whereas in cropland and orchards, stronger disturbance and lower organic inputs may promote a shift toward optical signatures of smaller apparent molecular size and lower aromaticity in deeper layers (Nakhavali et al., 2020; Zethof et al., 2019).

A notable exception was observed in some cropland sites such as millet and potato, where topsoil SUVA_254_ was relatively low but S_R_ was high, consistent with DOM enriched in low-aromatic fractions of smaller apparent molecular size. One likely explanation is that tillage and harvesting disturbances break aggregates and increase soil aeration, exposing previously protected organic matter to rapid decomposition (Weidhuner et al., 2021). At the same time, root exudates and microbial metabolites can accumulate near the surface and turn over quickly, and these products commonly exhibit low-aromatic optical signatures and small apparent molecular size. In contrast, more aromatic humic-like DOM may be selectively adsorbed and retained by clay and reactive minerals in the middle soil layer, causing a relative enrichment of aromatic structures and a local increase in SUVA_254_ at that depth (Fröberg et al., 2007; Helms et al., 2008).

The orchard species-m anagement contrast was also associated with DOM optical structure. In orchards, the apple/high-input site generally showed weaker aromatic optical signatures than the walnut/low-input site (*P* < 0.05), together with a smaller apparent molecular size in the topsoil, although not always significant (**Figures 9a**; **10a**). These differences may reflect stronger disturbance and lower surface cover at the apple/high-input site, together with species-specific litter chemistry (**Supplementary Figure 1d**), which may shorten DOM residence time in the topsoil and enhance vertical transport or rapid microbial consumption, leaving DOM with weaker aromatic signatures and smaller apparent molecular size. Walnut orchards, with less disturbance and more surface cover, may allow repeated microbial processing and stronger mineral interactions in the topsoil, resulting in stronger aromatic signatures and larger apparent molecular size (Knapen et al., 2007; Li et al., 2021). In grassland, we only detected a clear root-type effect in the subsoil, where taproot dominated sites showed higher S_R_ than fibrous-root sites (*P* < 0.05; **Figure 9a**), suggesting that deeper rooting may deliver more labile, smaller dissolved carbon compounds into deeper soil layers (Messing et al., 2003). In shrubland, artificially planted sites showed higher aromaticity than naturally restored sites in the middle and subsoil layers (*P* < 0.05; **Figure 10a**), while topsoil differences were small. This implies that artificial restoration may improve mid–deep soil conditions and promote microbial processing and stabilization of DOM, whereas in the topsoil, both restoration modes may converge due to generally high surface cover and BSCs development (Charamba et al., 2025; Riveras-Muñoz et al., 2022; Szyja et al., 2023).

Taken together, hypotheses (i) and (ii) were broadly supported: restored woody and grassland systems generally differed from cropland in DOC and optical composition, and DOM showed strong vertical differentiation. Hypothesis (iii) was partly supported. The orchard species-management contrast was clear for several indicators, whereas grassland root type and shrubland restoration mode produced weaker and indicator-dependent responses.

### 4.5 Study limitations and implications

This study establishes a field framework for interpreting soil DOM dynamics in drylands by integrating land use type, soil depth, and selected vegetation management contrasts. Unlike conventional land use comparisons, our design tracks DOM from topsoil to deeper layers while explicitly contrasting orchard management intensity, grassland root type, and shrubland restoration mode within the same sampling scheme. Using a consistent set of optical and fluorescence indicators and relating them to surface organic inputs and soil physical properties, we identify where land use effects are strongest along the profile and which factors most consistently explain DOM differences. These findings provide a practical basis for evaluating restoration effectiveness and optimizing dryland management by tracking changes in active carbon pools rather than relying only on bulk soil carbon.

Some limitations should be acknowledged. The study was conducted at representative sites with similar climate, topography, and soil-forming conditions, which helped reduce background variability but may limit extrapolation to other environmental settings. Because the sites represented existing field systems rather than a fully factorial experiment, species identity, vegetation traits, and management categories covaried; in particular, the apple/high-input and walnut/low-input orchards could not isolate management effects independently of tree species. These design features limit independent attribution of individual factors. In addition, interpretations were based mainly on optical and fluorescence indicators, which describe DOM composition but do not directly resolve the microbial and mineral processes controlling DOM transformation and stabilization. Moreover, DOM in dryland soils responds strongly to rainfall pulses, whereas the present sampling represents conditions within a limited time period and does not capture short-term hydrological effects.

Future work should extend observations across broader environmental gradients and integrate measurements of microbial activity, mineral composition, and soil structural properties. Seasonal sampling and event-based monitoring would further clarify DOM responses to rainfall variability and strengthen process-based interpretation. Such efforts will improve the use of DOM indicators in evaluating ecological restoration and land management in dryland systems.

## 5 Conclusion

Using DOC measurements, UV–Vis spectroscopy, EEM–PARAFAC, and multivariate analysis, this study examined profile-scale DOM across five land-use types and within-type contrasts in a typical dryland watershed of the hilly–gully Loess Plateau. Land use and soil depth structured DOM most strongly: forestland and shrubland had higher DOC contents and stronger humic-like and aromatic optical signatures, whereas deeper soils showed lower DOC contents and optical signatures consistent with greater microbial contributions. The coupled orchard tree species–management contrast was evident, whereas the effects of grassland root type and shrubland restoration mode were weaker and indicator-dependent.

These findings indicate that revegetation and subsequent management are associated with shifts in the quantity, composition, and vertical distribution of an active and mobile soil carbon pool. Maintaining surface cover and organic inputs while limiting unnecessary disturbance may help sustain DOM and humic-like optical characteristics during ecological restoration on the Loess Plateau, although this implication remains context-dependent. The conclusions most directly represent the investigated watershed and may inform comparable dryland settings. Future seasonal and event-based observations, together with microbial, mineralogical, and hydrological measurements, are needed to test their wider transferability.

## Data Availability

The datasets presented in this study can be found in online repositories. The names of the repository/repositories and accession number(s) can be found below: https://github.com/wangyicong031225-Lando/Research-data.git.
