# Supplementary material for: Soil dissolved organic matter dynamics under dryland restoration: disentangling land use, vegetation, and management controls
Source: Front Plant Sci. 2026 Aug 26;17:1934903. doi: 10.3389/fpls.2026.1934903 (PMC13553883; doi:10.3389/fpls.2026.1934903)
Supplement: Supplementary file 1 [file DataSheet1.docx]

**Soil dissolved organic matter dynamics under dryland restoration: disentangling land use, vegetation, and management controls**

Yi-cong Wang^a^, Qing-wei Zhang^a^*, Nan-yan Li, Yu-hui Zhang^a^, Guo Chen^b^, Dong-xu Wu^a^, Guo-xiong Gao^a^, Qiang Zhang^a^, Feng-bao Zhang^a^, Jian Wang^a^, Ming Li^c^, Hao Wang^a^

^a^ State Key Laboratory of Soil and Water Conservation and Desertification Control, , College of Soil and Water Conservation Science and Engineering, Northwest A&F University, Yangling, Shaanxi 712100, China

^b^ College of Urban and Environmental Sciences, Peking University, Beijing 100871, China

^c^ College of Natural Resources and Environment, Northwest A&F University, Yangling, Shaanxi 712100, China

*Corresponding author:

Email address: zhangqingwei@nwsuaf.edu.cn (Qing-wei Zhang)

TEL: +86-18700943518*

**Supplementary Information**

**1.1 Soil environmental factors**

Figure S1. Soil texture, BD, RD and LD across land use types in the (a) topsoil layer, (b) middle soil layer and (c) subsoil layer. (d) T_BSC_, C_BSC_, and (understory) vegetation coverage across the same land use types. The abbreviations, lowercase and uppercase letters on the bars follow the same conventions as in Fig. 2.

Figure S1 shows the soil environmental factors across all sites. RD peaked in the middle soil layer and was lowest in the topsoil layer, consistent with the typical depth distribution of most root systems. RD was also significantly higher in grassland and shrubland than in the other vegetation types (*P* < 0.05). In contrast, LD decreased with soil profile and was significantly higher in woody systems than in the other sites (*P* < 0.05). Both BSC thickness and coverage were highest in grassland, followed by forest land, and were significantly greater than those in shrubland and cropland (*P* < 0.05). Shrubland exhibited the highest understory vegetation coverage, and the understory layer in most sites was dominated by *Artemisia sacrorum* Ledeb.
